# HBV polymerase recruits the phosphatase PP1 to dephosphorylate HBc-Ser170 to complete encapsidation

**DOI:** 10.1371/journal.ppat.1012905

**Published:** 2025-02-11

**Authors:** Chi-Ling Hsieh, Li-Yang Chang, Pei-Jer Chen, Shiou-Hwei Yeh

**Affiliations:** 1 Graduate Institute of Clinical Medicine, National Taiwan University College of Medicine, Taipei, Taiwan; 2 Graduate Institute of Microbiology, National Taiwan University College of Medicine, Taipei, Taiwan; 3 Center of Precision Medicine, National Taiwan University, Taipei, Taiwan; 4 Department of Internal Medicine, National Taiwan University Hospital, Taipei, Taiwan; 5 Department of Clinical Laboratory Sciences and Medical Biotechnology, National Taiwan University College of Medicine, Taipei, Taiwan; University of Pittsburgh School of Medicine, UNITED STATES OF AMERICA

## Abstract

The HBV core (HBc) protein contains an N-terminal domain (NTD) for capsid assembly and an arginine-rich C-terminal domain (CTD) for pregenomic RNA (pgRNA) encapsidation. Phosphorylation of the HBc CTD, especially at Ser162 and Ser170, is essential for nucleation with the polymerase (Pol) to initiate pgRNA encapsidation. As capsids mature, the HBc CTD undergoes dephosphorylation, suggesting the involvement of a phosphatase in the late stage of encapsidation, which remains to be determined. Using a C-S170 antibody specific for non-phosphorylated HBc-Ser170, we observed a transition from a phosphorylated to a dephosphorylated state during pgRNA packaging. The Pol-dependent dephosphorylation of HBc-Ser170 was confirmed by the substitution of one single amino acid at Val782 in the RNase H domain, which abolished the dephosphorylation of HBc-Ser170. Immunoprecipitation, mass spectrometry analyses, and the protein structural analyses showed that the recruitment of the host phosphatase PP1 is dependent on the Pol-Val782 domain. This recruitment does not require HBc but does require Pol via epsilon RNA signal, suggesting that the Pol-pgRNA complex plays a key role in PP1 recruitment. Pol-pgRNA-PP1-mediated dephosphorylation of HBc-Ser170 is essential for the completion of pgRNA encapsidation and appears to be associated with late endosomes/multivesicular bodies (MVBs). Therefore, HBV Pol may play a dual role by initially bringing pgRNA to phosphorylated HBc and recruiting PP1 for later completion of RNA packaging into the capsids. These findings not only decipher the mechanism by which Pol-mediated dephosphorylation of HBc regulates pgRNA encapsulation, but also reveal the possibility of PP1 as a potential target for antiviral development.

## Author summary

Recent studies have emphasized the role of HBV core (HBc) protein in potential antiviral development because of its involvement in nucleocapsid assembly and pregenomic RNA (pgRNA) packaging. Our study focuses on the dephosphorylation of the HBc C-terminal domain (CTD) in regulating pgRNA encapsidation. Using an antibody specific for non-phosphorylated HBc-Ser170, a key epitope for pgRNA packaging, we observed a transition of HBc-Ser170 from a phosphorylated state to a dephosphorylated state during pgRNA encapsidation. We showed that dephosphorylation of HBc-Ser170 is dependent on the Val782 residue of Pol-RNase H domain, which is essential for recruitment of the host phosphatase PP1. Recruitment of PP1 by Pol-pgRNA complex facilitates the dephosphorylation of HBc-Ser170, which is crucial for the completion of RNA packaging into the viral capsids. These findings not only elucidate the sequence of events in pgRNA encapsidation, but also pave the way for the development of new HBV antiviral strategies targeting this mechanism.

## Introduction

Hepatitis B virus (HBV) is a hepatotropic virus with a 3.2 kb circular partially double-stranded DNA genome enclosed within an icosahedral nucleocapsid and outer envelope [1]. Following the infection of hepatocytes through the NTCP receptor and uncoating, the viral relaxed circular DNA (rcDNA) is imported and converted to closed circular DNA (cccDNA) in the nucleus [2]. This cccDNA serves as the template for the production of pregenomic RNA (pgRNA) and four viral mRNA transcripts, which are then translated into viral proteins [3]. Later, the HBV core (HBc) protein and polymerase (Pol) encapsidates the pgRNA to form the viral nucleocapsid, where reverse transcription and DNA synthesis occur [4,5]. Mature nucleocapsids containing rcDNA assemble with envelope proteins into complete virions that bud from multivesicular bodies (MVBs) [3,6]. The process of pgRNA encapsidation has emerged as a promising target for new antiviral therapies such as HBc protein allosteric modulators (CpAMs) [7]. Therefore, there has been substantial interest in understanding the mechanisms utilized by the HBc and Pol to execute pgRNA encapsidation.

The HBc protein, which is composed of ~183 a.a., is separated into an N-terminal domain (NTD, residues 1–140) and a C-terminal domain (CTD, residues 150–183) by a 10 a.a. linker sequence [8]. The HBc NTD is essential for capsid assembly *in vitro* [9,10], while the HBc CTD is responsible for the encapsidation of viral pgRNA [11,12]. The nucleic acid-binding activity of the HBc CTD is primarily attributed to positive charge from a series of four arginine-rich domains (ARDs) [13–16]. Previous studies have suggested that the charge balance of the HBc CTD is crucial for packaging pgRNA [15–17]. A decrease in the positive charge by Arg-to-Ala substitution in the CTD may reduce RNA packaging activity, which can however be rescued by decreasing negative charge by Ser-to-Ala substitution [18].

Interestingly, the pgRNA packaging activity of the HBc CTD can be affected by phosphorylation of the HBc CTD [16,19,20], which increases the negative charge of HBc. As documented, dimeric HBc and empty capsids exhibit hyperphosphorylated HBc, whereas nucleocapsids containing RNA or DNA exhibit hypophosphorylated HBc [21,22]. Among the seven putative phosphorylation sites identified in the CTD, Ser155, Ser162, and Ser170 are considered major phosphorylation sites [23,24]. Substitution of Ser162 or Ser170 with Ala significantly impaired viral pgRNA packaging [20,25]. However, substitution of S-to-E/D in these major Ser residues in the CTD also significantly reduces pgRNA packaging [19]. These studies indicated that the story is complicated and cannot be accounted for simply based on the charge balance theory.

It is possible that the phosphorylation of the HBc CTD is involved in the initial stage of pgRNA encapsidation, but the subsequent dephosphorylation of HBc is required for the enclosing of pgRNA into the mature capsid in the late stage of encapsidation. A putative phosphatase associated with the Pol-pgRNA complex has been proposed to perform this function. Although two phosphatases, PP2A and PP1, have been reported involved in the dephosphorylation of HBc [26,27], it remains unclear which phosphatase is responsible for this dephosphorylation process, how it is recruited into the Pol-pgRNA complex, and how it affects pgRNA encapsidation.

To address these questions, we generated an antibody (Ab) capable of distinguishing the phosphorylated and dephosphorylated forms of HBc at Ser170, which allowed us to monitor the phosphorylation pattern of HBc-Ser170 during the progressive stages of pgRNA packaging. Interestingly, we discovered that a unique domain of Pol is involved in the dephosphorylation of HBc-Ser170. Through this specific domain, HBV Pol recruits a host phosphatase, leading to the dephosphorylation of HBc-Ser170 for the enclosing of pgRNA in mature capsids. Our investigation of this molecular event elucidates the mechanism underlying the HBc phosphorylation-dephosphorylation cascade during pgRNA encapsidation into nucleocapsids.

## Results

### Generation of C-S170 Ab, which specifically recognizes the epitope of HBc containing non-phosphorylated Ser170

**Fig 1:**
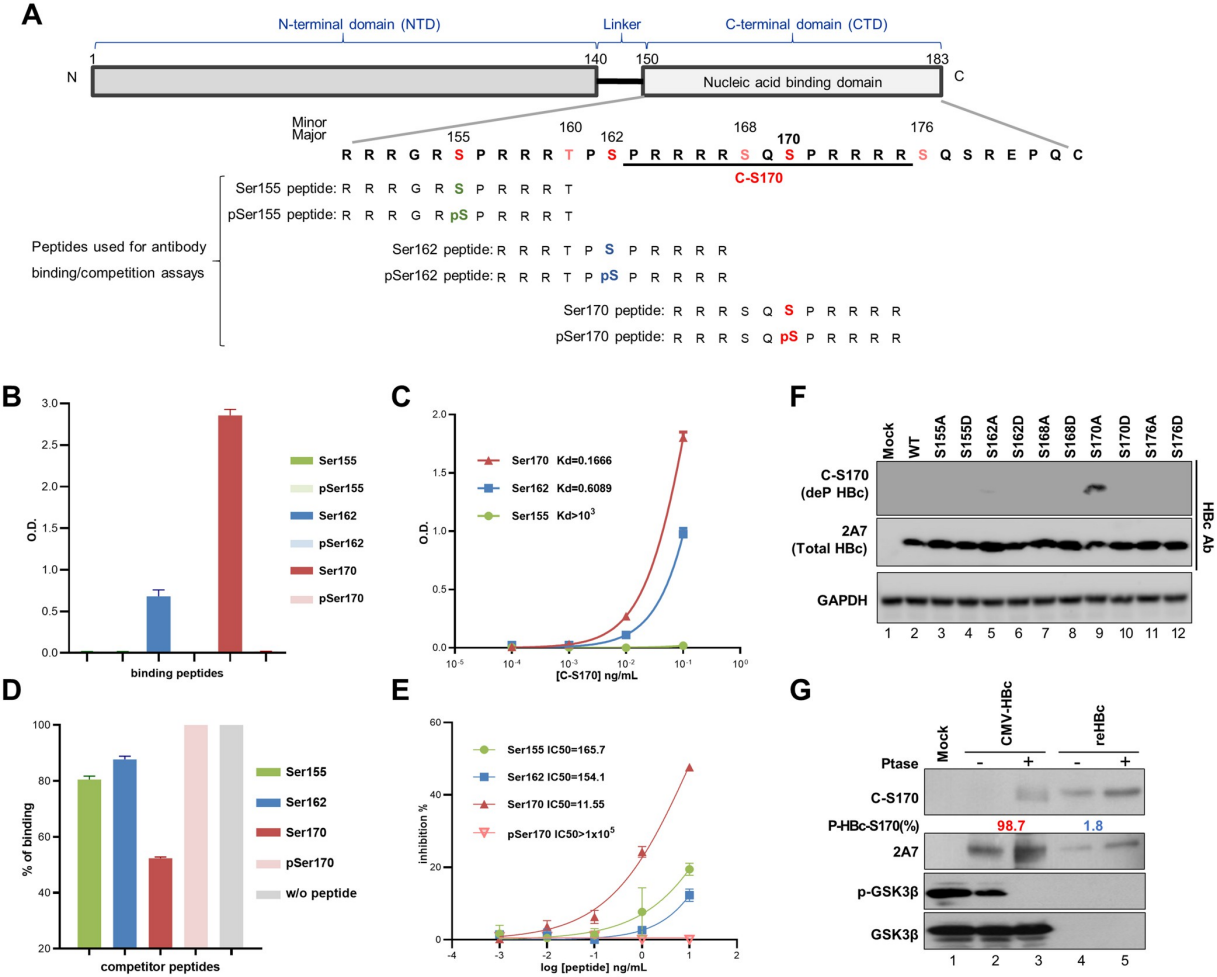
Specificity of the C-S170 Ab for non-phosphorylated HBc-Ser170. **(A)** Schematic diagram of the HBc sequence, highlighting the HBc CTD with indicated phosphorylation sites; epitopes recognized by C-S170 Ab are underlined. Peptides used for antibody binding/competition detection are shown at the bottom in relation to CTD sequences. **(B and C)** Binding of nonphospho- and phospho-HBc peptides covering each Ser residue to the C-S170 Ab. 100 ng of various peptides were immobilized on ELISA plates and tested for binding with C-S170 Ab. The binding affinity and kinetics for indicated peptides are represented in bar graphs with *Kd* values. **(D and E)** Competition of different HBc peptides for C-S170 Ab binding. Competition experiments were conducted in the presence of non-phosphorylated HBc-Ser170 peptide to evaluate the ability of indicated peptides to compete for C-S170 Ab binding. The competition kinetics is presented in bar graphs with IC_50_ values. **(F)** Specific recognition of the C-S170 Ab for non-phosphorylated HBc-Ser170. Huh7 cells were transfected with HBc expression constructs as indicated for 24 hr. Cell lysates were analyzed by immunoblotting, and hybridized with C-S170, 2A7, and GAPDH Abs. **(G)** Lysates of CMV-HBc-transfected Huh7 cells and E. coli recombinant HBc were treated with alkaline phosphatase (AP) prior to immunoblotting analysis. The percentage of phosphorylated HBc-Ser170 (P-HBc-S170%) was determined by comparing the adjusted C-S170 Ab signals with and without AP treatment, calculated as $1-\frac{adjusted\;C-S170\;signal\;\left(w/o\;Ptase\;treat\right)}{adjusted\;C-S170\;signal\;\left(w/\;Ptase\;treat\right)}$. Hyperphosphorylated HBc-S170 (>50%) is marked in red and hypophosphorylated HBc-S170 (<50%) is marked in blue.

To track the phosphorylation status of the HBc CTD during viral replication and maturation, we have tried to generate Abs capable of distinguishing between the phosphorylated and dephosphorylated forms of the HBc CTD. We successfully generated a polyclonal C-S170 Ab that specifically recognizes one epitope in HBc containing non-phosphorylated Ser170 (Fig 1A). To confirm its specificity, we conducted an *in vitro* binding assay using a panel of synthetic peptides covering Ser155, Ser162, and Ser170 (Fig 1A). The ELISA results showed that the C-S170 Ab had the highest binding affinity for the non-phosphorylated peptide covering Ser170 (Fig 1B). Consistently, the dissociation constant (*Kd*) values demonstrated that C-S170 had the greatest capacity to interact with the peptide containing non-phosphorylated Ser170 (Fig 1C). Moreover, competition binding assays revealed that the peptide containing non-phosphorylated Ser170 had the greatest ability to compete for binding, while the presence of phosphorylated Ser170 peptide had no effect on binding (Fig 1D and 1E). These results strongly supported the specificity of the C-S170 Ab in recognizing the epitope containing non-phosphorylated Ser170 in HBc.

In addition to experiments involving these synthetic peptides, we validated the recognition pattern of the C-S170 Ab for full-length HBc. Wild-type or mutant HBc expression constructs, including mutants with Ala and Asp substitutions at Ser155, Ser162, Ser168, Ser170, and Ser176, were transfected into Huh7 cells. Cell lysates were harvested for immunoblotting analysis using C-S170 Ab and control anti-HBc Ab (2A7 Ab for targeting a.a. 140–152). All forms of HBc, including wild-type and mutant HBc, were well recognized by 2A7 Ab (Fig 1F). However, only the S170A mutant was recognized by the C-S170 Ab (Fig 1F, lane 9), supporting the specificity of the C-S170 Ab for recognizing epitopes containing non-phosphorylated Ser170. Moreover, the predominant form of HBc in the cells was phosphorylated at Ser170, as indicated by the lack of a detectable signal of C-S170 Ab (Fig 1F, lane 2). To further verify this finding, lysates from CMV-HBc transfected cells and bacteria-expressed recombinant HBc (reHBc) were treated with alkaline phosphatase (AP) for immunoblotting analysis. Only AP-treated CMV-HBc showed an increase in the C-S170 Ab recognition signal (Fig 1G, lanes 2–3). In contrast, treatment of reHBc (almost exclusively in dephosphorylated form) with AP had no effect on the C-S170 Ab recognition signal (Fig 1G, lanes 4–5). These results confirmed that the C-S170 Ab preferentially recognizes HBc containing dephosphorylated Ser170.

### HBc transitions from a form containing phosphorylated Ser170 in incomplete capsids to one containing dephosphorylated Ser170 in complete capsids with pgRNA or DNA

Aided by the C-S170 Ab, we conducted immunoblotting analysis to investigate the phosphorylation status of HBc at Ser170 at progressive stages of capsid assembly, including the HBc dimer, incomplete capsids, and complete capsids. We used the “replication stage arrest approach” by introducing a specific Pol mutation to halt replication at a specific step to enrich specific replication intermediates for analysis [28–30]. The precore-null (Pc^-^) replicon was used as a template for site-directed mutagenesis to avoid interference from the processed precore protein [31].

The C-Y132A mutant only forms dimers, and this mutation halts further HBc assembly [28]. The Pol protein is responsible for carrying pgRNA to the capsid, leading to pgRNA encapsidation [32]. The Pol null (Pol^-^) construct completely lacks the capacity for pgRNA encapsidation, resulting in the accumulation of empty capsids. The Pol-Y65D mutant is defective in the primase domain, and this mutation allows for the packaging of pgRNA but impairs the conversion of pgRNA to DNA, resulting in the accumulation of pgRNA-containing capsids [30]. The Pol-E731H mutant, which is defective in RNase H activity, which is necessary for removing pgRNA to facilitate plus-strand DNA synthesis, resulting in the accumulation of pgRNA/ssDNA-containing capsids [33]. The surface null (LMS^-^) construct was included to enrich mature capsids before envelopment and secretion [34]. The mutations introduced in each of the mutant replicon constructs are shown in S1 Fig. Because of the multiple overlapping nature of the HBV translation frames, special attention was taken in designing these mutations to affect only the gene to be mutated and no other viral genes in most cases (S1 Fig). All of these mutations were sequence-validated.

These constructs were transfected into Huh7 cells and the enrichment of specific HBV replication intermediates (outlined in S2A Fig), along with the presence of encapsidated RNA or reverse-transcribed DNA at the expected replication stages, was validated by native agarose gel electrophoresis (for capsid assembly and encapsidated DNA) and Northern blotting (for encapsidated RNA) as appropriate (S2B Fig). The presence of both empty virions and genome-containing virions in the supernatant, separated by sucrose density gradient, was confirmed using native agarose gel electrophoresis and qPCR (S2C Fig).

**Fig 2:**
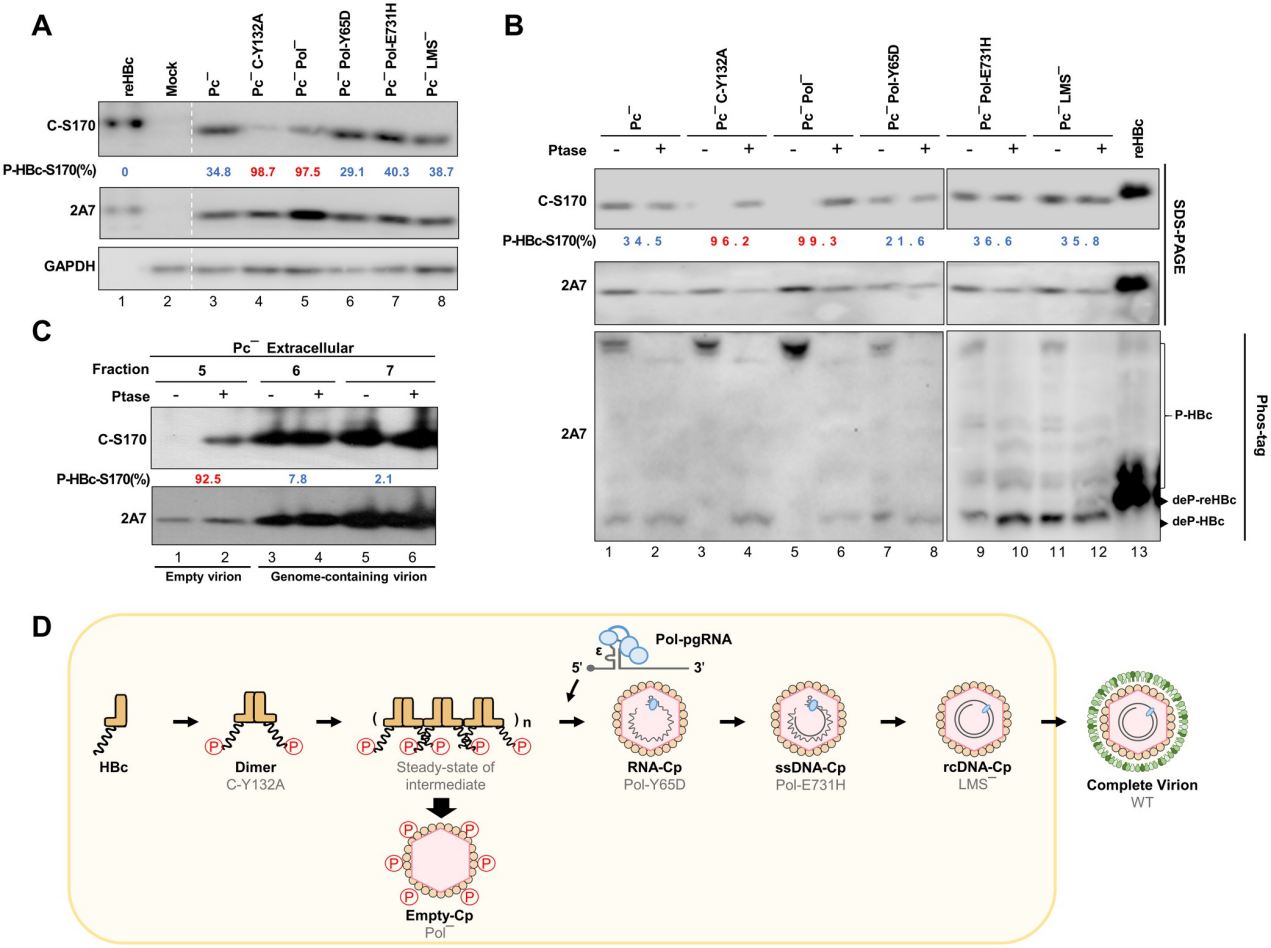
The phosphorylation status of HBc-Ser170 changes from phosphorylation to dephosphorylation during RNA encapsidation step. **(A)** Phosphorylation pattern of HBc in different HBV replicons. Huh7 cells were transfected with indicated HBV replicon constructs for 24 hr and the phosphorylation pattern was analyzed using the C-S170 signal, calculated as $1-\frac{adjusted\;C-S170\;signal\;\left(mutant\right)}{adjusted\;C-S170\;signal\;\left(reHBc\;control\right)}$. The reHBc was used as the dephosphorylated-HBc control. **(B)** Lysates of HBV replicon-transfected Huh7 cells were treated with AP prior to SDS-PAGE and Phos-tag SDS-PAGE analysis. The reHBc was used as the dephosphorylated-HBc control. (Upper panel) P-HBc-S170% was determined by comparing C-S170 Ab signals with and without AP treatment, calculated as $1-\frac{adjusted\;C-S170\;signal\;\left(w/o\;Ptase\;treat\right)}{adjusted\;C-S170\;signal\;\left(w/\;Ptase\;treat\right)}$, provided that the C-S170/2A7 ratios for different groups of AP-treated HBc and reHBc are close to each other (<15% difference). Hyperphosphorylated HBc-S170 (>50%) is marked in red and hypophosphorylated HBc-S170 (<50%) is marked in blue. (Lower panel) Lysates were analyzed by Phos-tag SDS-PAGE and the positions of phosphorylated-HBc and dephosphorylated-HBc or dephosphorylated-reHBc are indicated. **(C)** Virions from the supernatant of Huh7 cells transfected with different HBV replicons were precipitated by sucrose cushion centrifugation. The empty virions and genome-containing virions were separated by sucrose density gradient and the phosphorylation status was analyzed by AP assay. Fraction 5 was loaded at half the lysate amount of fractions 6 and 7. **(D)** Schematic illustration of the dynamic changes in HBc-Ser170 phosphorylation at different viral replication steps, with a transition from phosphorylated to dephosphorylated HBc-Ser170 at pgRNA encapsidation step.

To investigate the phosphorylation status of HBc at Ser170, protein lysates harvested from these transfected cells were processed for SDS-PAGE and Phos-tag SDS-PAGE with the 2A7 Ab (total HBc) and C-S170 Ab (non-phosphorylated HBc), either without (Fig 2A) or with AP treatment (Fig 2B). Meanwhile, we added recombinant HBc (reHBc) as an internal control (fully dephosphorylated) to help evaluate the phosphorylation level of HBc in the transfected cells of each replicon.

The results showed that the C-S170 signals of C-Y132A (dimer) and Pol^-^ (empty capsid) were significantly lower than those of Pc^-^ transfected cells; whereas the C-S170 signals were significantly increased after AP treatment, suggesting that HBc-Ser170 was predominantly hyperphosphorylated (Fig 2A, lanes 4–5; Fig 2B, lanes 3–6). In contrast, the C-S170 signals for the replicons able to form genome-containing capsids, such as Pol-Y65D (pgRNA), Pol-E731H (pgRNA/ssDNA) and LMS^-^ (rcDNA), had comparable high levels as Pc^-^, and remained high with little change upon AP treatment, indicating that HBc mainly exhibited the hypophosphorylated form (Fig 2A, lanes 6–8; Fig 2B, lanes 7–12). The C-S170/2A7 ratios of the different groups of AP-treated cell lysates showed relatively small differences compared to reHBc, suggesting that AP treatment was effective; the percentage of phosphorylated HBc-Ser170 in each replicon was then calculated accordingly (Fig 2A and 2B). Consistent with this, the results of Phos-tag gel electrophoresis showed that C-Y132A and Pol^-^ expressed almost fully phosphorylated HBc, whereas the other constructs expressed phosphorylated and dephosphorylated HBc (Fig 2B, lower panel).

The AP analysis was also applied to HBc in different fractions from sucrose density gradient analysis of the extracellular supernatants (S2C Fig). HBc in the fractions containing genome-containing virions (fractions 6–7) was largely hypophosphorylated (Fig 2C, lanes 3–6), whereas that in the fraction containing empty virions (fraction 5) was hyperphosphorylated (Fig 2C, lanes 1–2).

The dynamic changes in the phosphorylation status of Ser170 in HBc at progressive replication stages are schematically summarized in Fig 2D. These results indicated a transition from phosphorylated to dephosphorylated Ser170 in HBc at the RNA encapsidation stage, consistent with a previous publication [22], suggesting that the dephosphorylation of HBc at Ser170 might play an important role in the complete packaging of HBV pgRNA.

### Critical role of the residue Val782 in Pol in regulating HBc-Ser170 dephosphorylation and HBV replication

**Fig 3:**
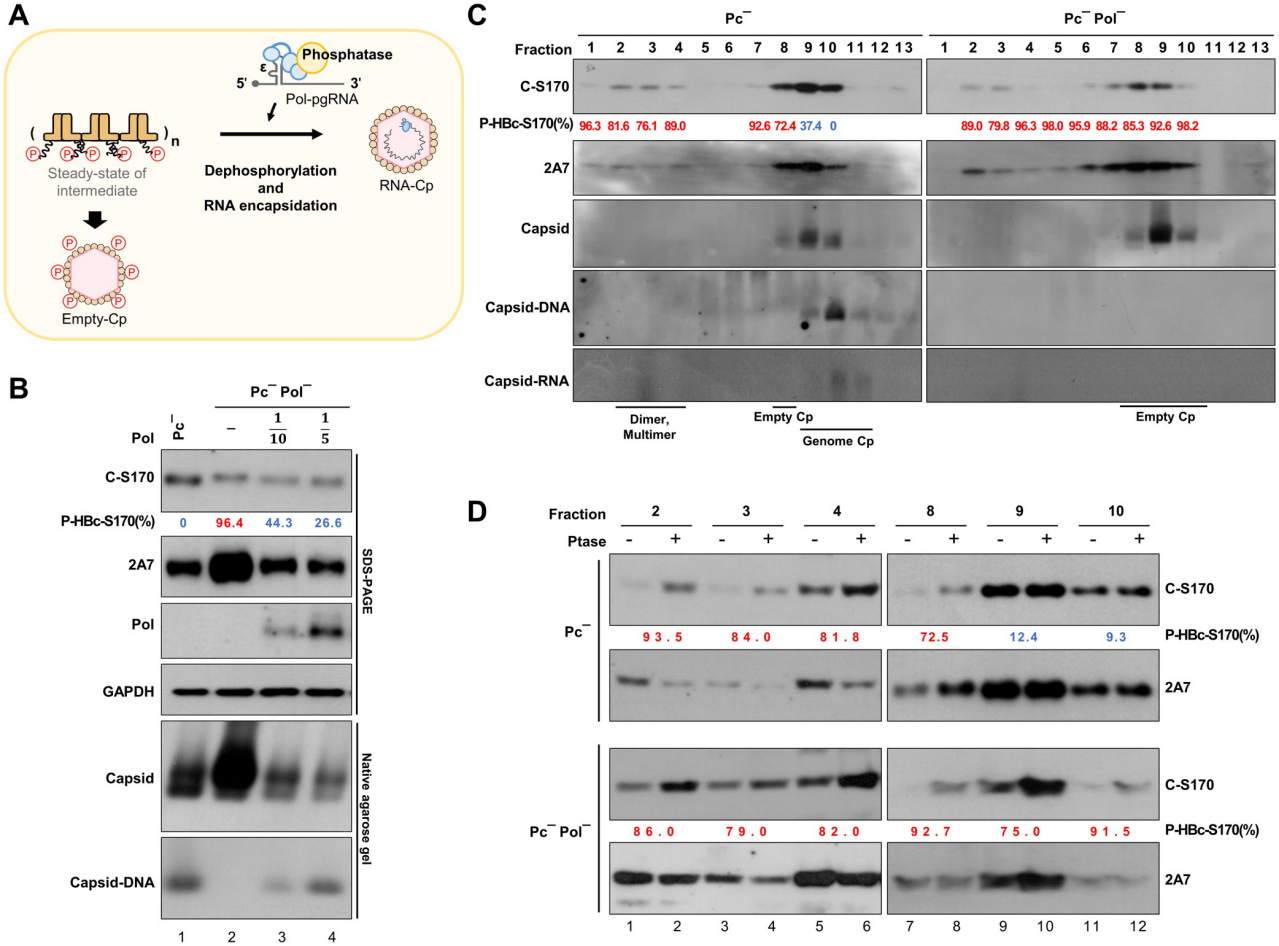
Pol-dependent HBc-Ser170 dephosphorylation is critical for pgRNA encapsidation. **(A)** Schematic illustration of the phosphorylation to dephosphorylation of HBc-Ser170 during RNA encapsidation, suggesting that a phosphatase is involved in the Pol-dependent dephosphorylation of HBc-Ser170. **(B)** Pol-dependent HBc-Ser170 dephosphorylation and pgRNA encapsidation. Huh7 cells were cotransfected with HBV replicon and Pol constructs as indicated for 24 hr. The protein lysate was analyzed by immunoblotting; the capsids and capsid DNA were analyzed by native agarose gel electrophoresis and Southern blotting. The P-HBc-S170% of indicated experimental set was determined by $1-\frac{adjusted\;C-S170\;signal\;\left(exp.set\right)}{adjusted\;C-S170\;signal\;\left(Pc^{-}\;control\right)}$. **(C)** Huh7 cells were transfected with indicated HBV replicon constructs for 48 hr, followed by sucrose density gradient analysis to separate HBc dimers/multimers and capsids. The protein content, capsids, capsid DNA, and capsid RNA in each fraction were analyzed by immunoblotting, native agarose gel electrophoresis, Southern blotting, and Northern blotting, respectively. The P-HBc-S170% of indicated fractions was determined by $1-\frac{adjusted\;C-S170\;signal\;\left(indicated\;fraction\right)}{adjusted\;C-S170\;signal\;(Pc^{-}\;fraction\;10)}$. **(D)** Phosphorylation pattern of the HBc dimer and capsids. The phosphorylation status of HBc in fractions containing either HBc dimer or capsids as indicated was analyzed by AP assay. The P-HBc-S170% of indicated fractions was validated by AP analysis, determined by $1-\frac{adjusted\;C-S170\;signal\;\left(w/o\;Ptase\;treat\right)}{adjusted\;C-S170\;signal\;\left(w/\;Ptase\;treat\right)}$. Hyperphosphorylated HBc-S170 (>50%) is marked in red and hypophosphorylated HBc-S170 (<50%) is marked in blue.

The dephosphorylation of HBc-Ser170 coincided with the encapsidation of RNA, suggesting that the viral Pol protein may act as a carrier for this putative phosphatase (Fig 3A). To assess the effect of Pol on HBc-Ser170 dephosphorylation, we performed a Pol complementation assay. The results showed that the Pol^-^ replicon was unable to dephosphorylate HBc-Ser170 and package nucleic acids (Fig 3B, lane 2), but supplementation with Pol alleviated both effects in a dose-dependent manner (Fig 3B, lanes 3–4). In addition, we found that supplementation with Pol significantly reduced HBc expression (Fig 3B, lanes 3–4), which may be due to competition for translation of the pgRNA template as a result of Pol binding to the 5’-epsilon of pgRNA [35,36]. To further examine the effect of Pol on the dephosphorylation of HBc capsids, sucrose density gradient was used to separate the incomplete and complete capsids in cells transfected with the Pol(+) or Pol(-) HBV replicon, including dimers/multimers (fractions 2–4), empty capsids (fraction 8) and genome-containing capsids (fractions 9–10) (Fig 3C). Although our sucrose gradient analysis was not able to completely separate empty capsids from genome-containing capsids (fractions 8–10), we still found a higher proportion of hypophosphorylated form of HBc in genome-containing capsids by the presence of Pol (Fig 3C and 3D; fractions 9–10). In contrast, the phosphorylation of both multimeric and empty capsids was unaffected by the presence of Pol and remained hyperphosphorylated (Fig 3C and 3D, fractions 2–4, 8). These findings suggested that Pol may play a role in the transition of the HBc phosphorylation status from hyperphosphorylated to hypophosphorylated during the completion of pgRNA packaging.

To further investigate the mechanism of Pol-mediated HBc-Ser170 dephosphorylation, we identified the domain in Pol that is responsible for its dephosphorylation function. We generated several point mutations in the three domains of Pol: the TP domain, reverse transcriptase (RT) domain and RNase H domain (S1 Fig) [32,33,37]. Pol-Y65D is mutant of the TP domain [30], Pol-YMHD and Pol-YMHA are mutants of the reverse transcriptase YMDD motif [37], and Pol-E731H and Pol-V782Y are mutants of RNase H domain. Pol-V782Y was selected because it is conserved among all hepadnaviruses and was initially shown to have an effect on duck HBV (DHBV) replication [33].

**Fig 4:**
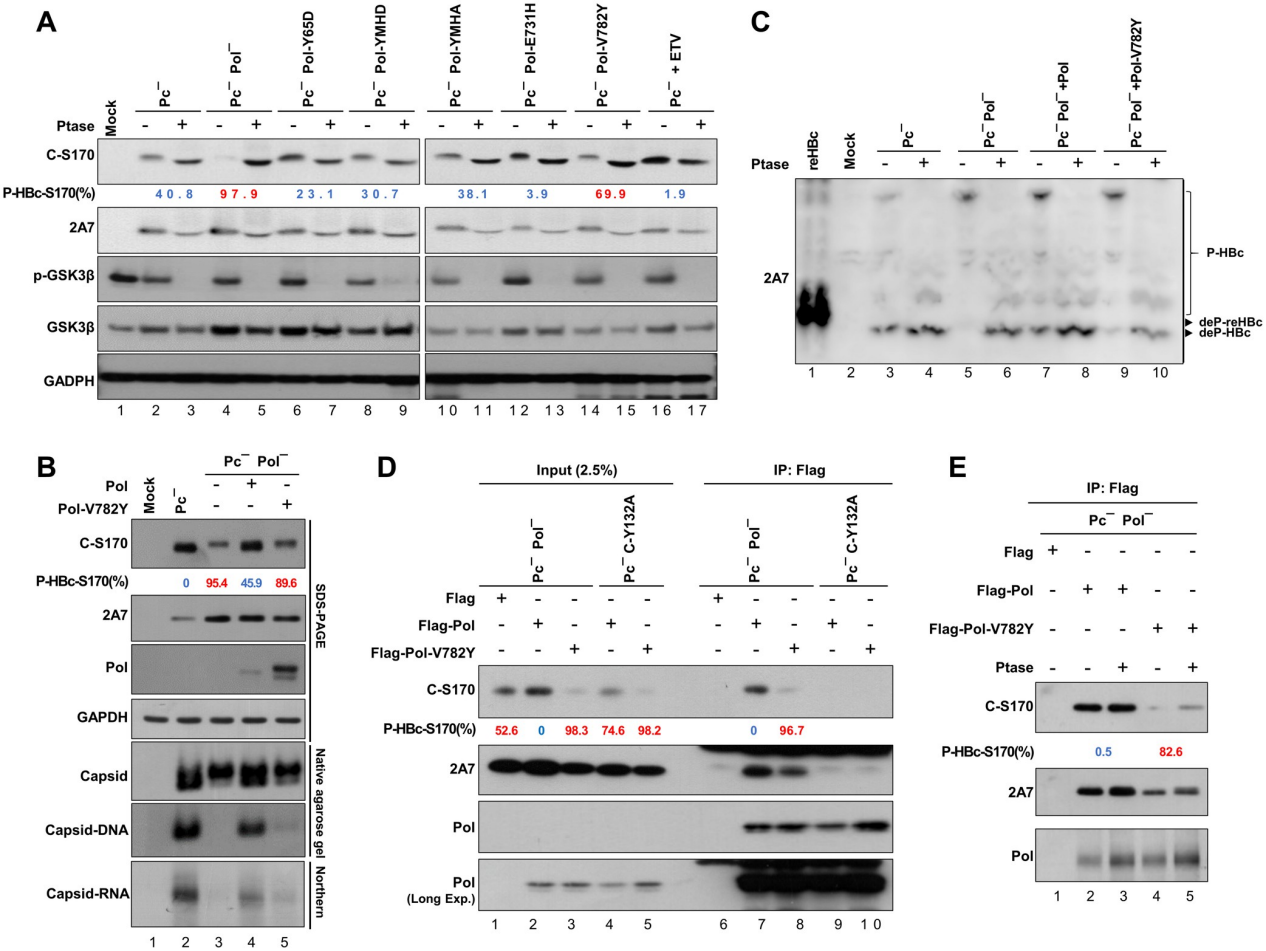
The phosphatase-associated domain at Pol-Val782 recruits phosphatase to dephosphorylate HBc-Ser170. **(A)** HBc phosphorylation status in HBV Pol mutant replicons. Huh7 cells were transfected with HBV replicon constructs as indicated for 24 hr. The protein lysates were processed for immunoblotting. The P-HBc-S170% was validated by AP analysis, determined by $1-\frac{adjusted\;C-S170\;signal\;\left(w/o\;Ptase\;treat\right)}{adjusted\;C-S170\;signal\;\left(w/\;Ptase\;treat\right)}$. **(B)** Pol-Val782-dependent HBc-Ser170 dephosphorylation and pgRNA encapsidation. Huh7 cells were cotransfected with HBV replicon and Pol constructs at a 20:1 replicon:Pol ratio for 24 hr. The protein content, capsids, capsid DNA, and capsid RNA were analyzed by immunoblotting, native agarose gel electrophoresis and Northern blotting analysis, respectively. The P-HBc-S170% of indicated experimental set was determined by $1-\frac{adjusted\;C-S170\;signal\;\left(exp.set\right)}{adjusted\;C-S170\;signal\;(Pc^{-}\;control)}$. **(C)** Huh7 cells were cotransfected with HBV replicon and Pol constructs as indicated for 24 hr. Phosphorylation patterns of HBc were analyzed by Phos-tag SDS-PAGE with AP treatment. The reHBc was used as the dephosphorylated-HBc control. **(D and E)** Phosphorylation status of Pol-interacting HBc. Huh7 cells were cotransfected with the HBV replicon and Flag-Pol constructs at a 20:1 replicon:Pol ratio for 24 hr. Pol and Pol-interacting HBc were immunoprecipitated with anti-Flag Ab, and the phosphorylation pattern of HBc was analyzed by comparing the signals of C-S170 Ab with 2A7 Ab and validated by AP assay. The P-HBc-S170% of indicated experimental set was determined by $1-\frac{adjusted\;C-S170\;signal\;\left(exp.set\right)}{adjusted\;C-S170\;signal\;(Pc^{-}Pol^{-}+Flag-Pol\;)}$. The P-HBc-S170% of indicated experimental set was validated by AP analysis, determined by $1-\frac{adjusted\;C-S170\;signal\;\left(w/o\;Ptase\;treat\right)}{adjusted\;C-S170\;signal\;\left(w/\;Ptase\;treat\right)}$. Hyperphosphorylated HBc-S170 (>50%) is marked in red and hypophosphorylated HBc-S170 (<50%) is marked in blue.

Similar to the Pol-WT replicon, HBc-Ser170 remained hypophosphorylated in the Pol mutant replicons Pol-Y65D, YMHD, YMHA, and E731H (Fig 4A). However, HBc-Ser170 was hyperphosphorylated in the Pol-V782Y mutant replicon (Fig 4A, lanes 14–15), suggesting the importance of the residue Val782 in Pol for HBc dephosphorylation. We then conducted a complementation assay to confirm that Pol-V782Y was unable to dephosphorylate HBc-Ser170. While HBc-Ser170 dephosphorylation was restored by Pol-WT, complementation with Pol-V782Y did not lead to the dephosphorylation of HBc-Ser170 (Fig 4B, lane 5). Moreover, Phos-tag analysis showed that whereas only phosphorylated HBc was present in Pc^-^Pol^-^ expressing cells, complementation of WT-Pol but not Pol-V782Y resulted in dephosphorylation of HBc in Pc^-^Pol^-^ expressing cells (Fig 4C, lanes 7–8 vs. 9–10). In addition, we found that hyperphosphorylation of HBc-Ser170 in the Pol-V782Y replicon was associated with a significant reduction in pgRNA content in the capsids (Fig 4B, lane 5).

To investigate the role of the residue Val782 in Pol in regulating the dephosphorylation of HBc-Ser170 and RNA encapsidation, we conducted co-immunoprecipitation (co-IP) to evaluate the impact of Flag-Pol-V782Y on the interaction between HBc and Flag-Pol. Flag-Pol-V782Y exhibited a HBc-binding capacity similar to that of Flag-Pol (Fig 4D); however, the HBc associated with Flag-Pol-V782Y failed to undergo dephosphorylation at Ser170 (Fig 4D, lane 8; Fig 4E, lane 4–5). Our results also showed that the HBc dimer from the Pc^-^ C-Y132A replicon, which was used as a control, did not sufficiently interact with Flag-Pol (Fig 4D, lanes 9–10). Taken together, our co-IP experiments showed that Pol interacted with HBc in incompletely assembled capsids rather than HBc in nucleocapsids, since the antibody cannot access Pol once it was assembled into the capsids. The results thus suggested that dephosphorylation of HBc-Ser170 by Pol-Val782 occurs in incompletely assembled capsids, which may be due to the activity of the phosphatase associated with Pol-Val782.

### Pol-Val782-dependent dephosphorylation of HBc-Ser170 occurs via recruitment of the catalytic subunit of the phosphatase PP1 to late endosomes/MVBs

**Fig 5:**
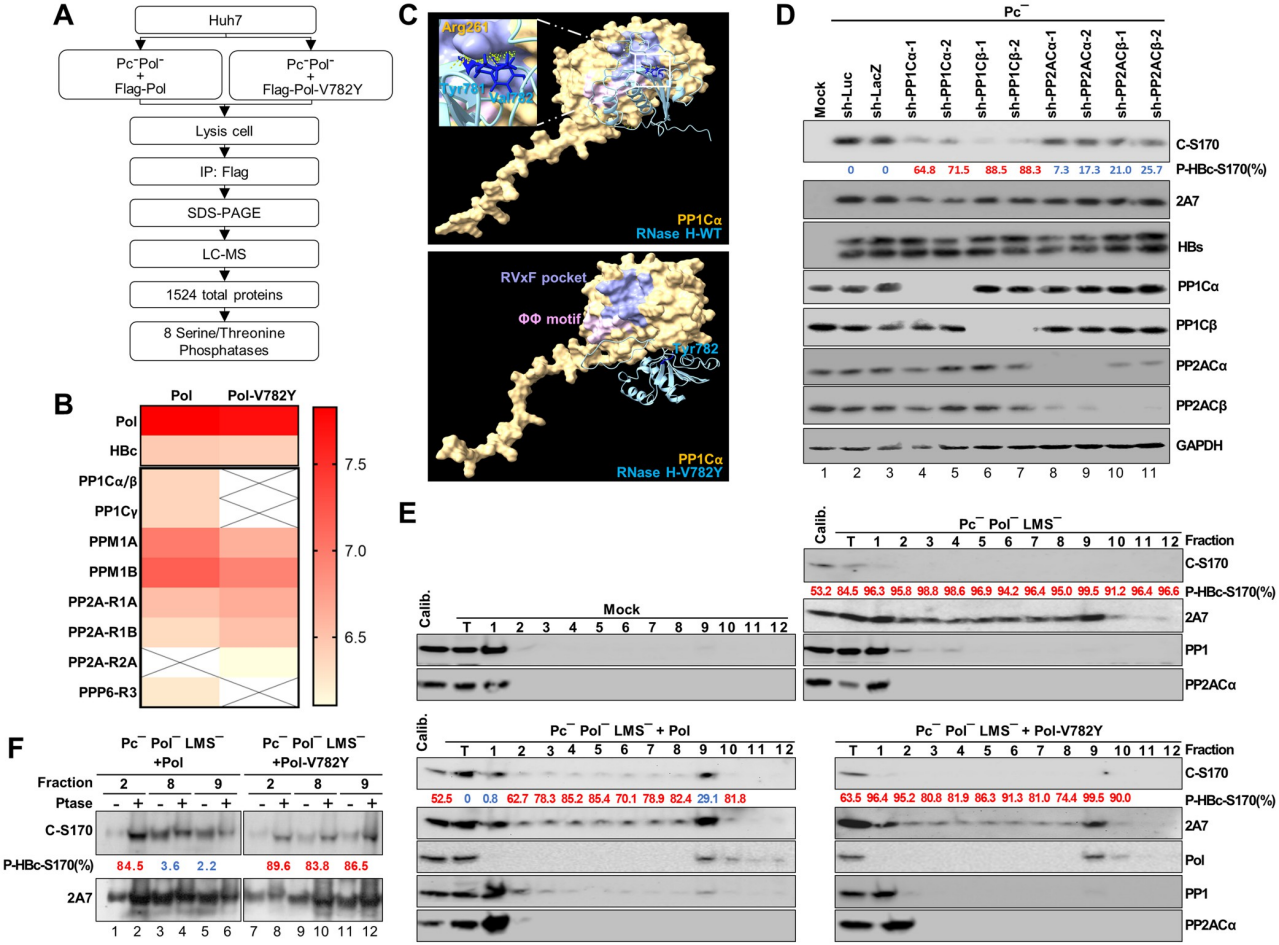
PP1 dephosphorylates HBc-Ser170 at late endosomes/MVBs through Pol recruitment. **(A and B)** Pol-interacting phosphatases identified via IP/mass spectrometry. Huh7 cells were cotransfected with the HBV replicon and Flag-Pol constructs as indicated for 24 hr. Pol-interacting proteins were coimmunoprecipitated and eluted via SDS-PAGE for LC-MS analysis. MS data were filtered to identify Pol-interacting phosphatases. **(C)** Predicted model of Pol-RNase H domain and PP1Cα interaction. Cartoon representation of the structures of Pol-RNase H (blue) and PP1Cα (light yellow). The RVxF binding pocket is highlighted in lavender, and the ΦΦ binding motif is marked with pink. The model focused on the interactions between Pol RNase H residues Tyr781 and Val782 and the PP1Cα residue Arg261. Atom-atom contacts are represented by yellow dashed lines. **(D)** Validation of PP1 dephosphorylation of HBc-Ser170. Huh7 cells were transfected with HBV replicon construct after knockdown of specific phosphatase gene as indicated for 24 hr, and then processed for immunoblotting analysis. The P-HBc-S170% of indicated experimental set was determined by $1-\frac{adjusted\;C-S170\;signal\;\left(exp.set\right)}{adjusted\;C-S170\;signal\;\left(Pc^{-}+shLuc\right)}$. **(E)** Distribution of PP1 in cells expressing different HBV replicons. Huh7 cells were co-transfected with HBV replicon and Pol constructs as indicated for 48 hr. Cell compartments were separated using a sucrose density gradient for cell fractionation and analyzed by immunoblotting analysis. The P-HBc-S170% of indicated fractions was determined by $1-\frac{adjusted\;C-S170\;signal\;\left(indicated\;fraction\right)}{adjusted\;C-S170\;signal\;\left(Pc^{-}Pol^{-}LMS^{-}+Pol\right)}$. Calibrator: an aliquot of lysate from Pc^-^ Pol^-^ LMS^-^ + Pol-V782Y transfected cells. **(F)** The phosphorylation status of PP1-associated HBc in PP1-containing fractions was validated by the AP assay. The P-HBc-S170% of indicated fractions was determined by $1-\frac{adjusted\;C-S170\;signal\;\left(w/o\;Ptase\;treat\right)}{adjusted\;C-S170\;signal\;\left(w/\;Ptase\;treat\right)}$. Hyperphosphorylated HBc-S170 (>50%) is marked in red and hypophosphorylated HBc-S170 (<50%) is marked in blue.

Previous studies have shown that two phosphatases, PP1 and PP2A, may contribute to the dephosphorylation of HBc during HBV replication [26,27]. To identify the phosphatase that is associated with Pol during RNA enclosure, we expressed Flag-Pol-WT and Flag-Pol-V782Y in the Pc^-^Pol^-^ replicon and performed IP-mass spectrometry to identify any phosphatase that binds exclusively to Pol-WT and not to Pol-V782Y (Fig 5A). A total of eight serine/threonine phosphatases that associated with either Pol-WT or Pol-V782Y were identified (Fig 5B, S1 Table). Among them, PP1 selectively interacted with Pol-WT but not Pol-V782Y. On the other hand, PP2A showed no discernible difference in its binding to Pol-WT or to Pol-V782Y (Fig 5B, S1 Table). We further compared the PP1 expression patterns between empty capsids (fraction 8) and genome-containing capsids (fractions 9–10) from Pc^-^ and in Pc^-^ Pol^-^ expressing cells (Fig 3C). The results showed that PP1 was present in genome-containing capsid fractions (S3A Fig, lanes 2–3) but not in the empty capsid fractions (S3A Fig, lanes 1, 4–6). In addition, co-IP analysis showed that PP1 could co-IP with HBc Ab (S3B Fig, lane 8), while HBc could not co-IP with PP1 Ab (S3B Fig, lane 11). These results indicated that PP1 is encapsidated in the Pol-pgRNA containing capsids, which is consistent with the previous study by Hu Z et al [26].

In support of this finding, we developed a predictive model that provides insights into the proposed interactions between the Pol-RNase H domain and PP1Cα. This model specifically focuses on the intricate interactions between the residues Tyr781 and Val782 in Pol-RNase H and the residue Arg261 in the RVxF pocket of PP1Cα, a well-known regulator docking pocket on PP1C (Fig 5C, upper panel) [38]. and these interactions were abolished when Pol-Val782 was changed to Pol-Tyr782 (Fig 5C, lower panel). In addition, the protein prediction model did not reveal an equivalent interaction between Pol and PP2A through Pol-Val782 (S4 Fig).

To examine the dephosphorylation of HBc-Ser170 by PP1, we conducted a PP1 knockdown experiment. Knockdown of PP1Cα or PP1Cβ increased the phosphorylation of HBc-Ser170, while knockdown of PP2ACα or PP2ACβ did not change the HBc-Ser170 phosphorylation status (Fig 5D). Furthermore, we used sucrose density gradient for cell fractionation to separate different cellular compartments [39] to verify the colocalization of Pol and PP1. In this analysis, the cytosol and ER were present in fractions 1 and 2 (S5 Fig), while late endosomes/MVBs were present in fraction 9 (S5 Fig). HBc in the cytosol was suggested to undergo trafficking via autophagy, in which an autophagosome fuses with MVBs to form mature virions with envelope proteins [40]. Indeed, our data also revealed that HBc was distributed in the cytosol, ER, and late endosomes/MVBs, while Pol was predominantly localized in only late endosomes/MVBs (Figs 5E and S6D right panel). In the absence of Pol, PP1 was primarily distributed in the cytosol and ER. However, PP1 translocated to late endosomes/MVBs by complementation with Pol-WT but not with the mutant Pol-V782Y (Fig 5E, fraction 9 and S6D Fig right panel).

We next tried to address whether Pol-dependent HBc-Ser170 dephosphorylation was dependent on other viral factors, such as HBc or pgRNA, by the same sucrose gradient-based compartmentation analysis, First, we expressed Flag-Pol or Flag-Pol-V782Y in Huh7 cells, but did not find changes in the distribution of endogenous PP1 to the Pol-containing fractions (S6A Fig, fractions 9–10). The results suggested that the recruitment of PP1 by Pol needs some other viral components. To further identify key viral factors, we expressed Pol as well as HBV replicons either without HBc (Ec^-^ Pol^-^, lack of HBc and Pol expression by prestop mutation) or devoid of interactions between Pol and pgRNA (Pc^-^ ε-B1G Pol^-^, with a mutation at the epsilon RNA signal abolishing Pol binding; S6C Fig, left panel) [41] for the compartmentation assay. The results showed that PP1 can be recruited to the Pol-containing fractions (fractions 9–10, late endosomes/MVBs) even in the absence of HBc (S6B Fig), which however did not occur in the absence of Pol-pgRNA complex (S6D Fig). The results thus suggested Pol-dependent recruitment of PP1 depended on pgRNA through the epsilon RNA signal. Additionally, HBc-Ser170 dephosphorylation also depended on PP1 recruitment by this Pol-pgRNA complex (S6C Fig, right panel), consistent with previous studies [42]. Furthermore, we detected dephosphorylated HBc-Ser170 in late endosomes/MVBs, which was mediated by Pol-recruited PP1. In contrast, the presence of Pol-V782Y did not result in the dephosphorylation of HBc-Ser170 in late endosomes/MVBs (Fig 5F).

These results collectively suggest that the residue Val782 in Pol plays a crucial role in facilitating the recruitment of PP1 to dephosphorylate HBc-Ser170.

### HBc dephosphorylation contributes to RNA enclosure into capsids

**Fig 6:**
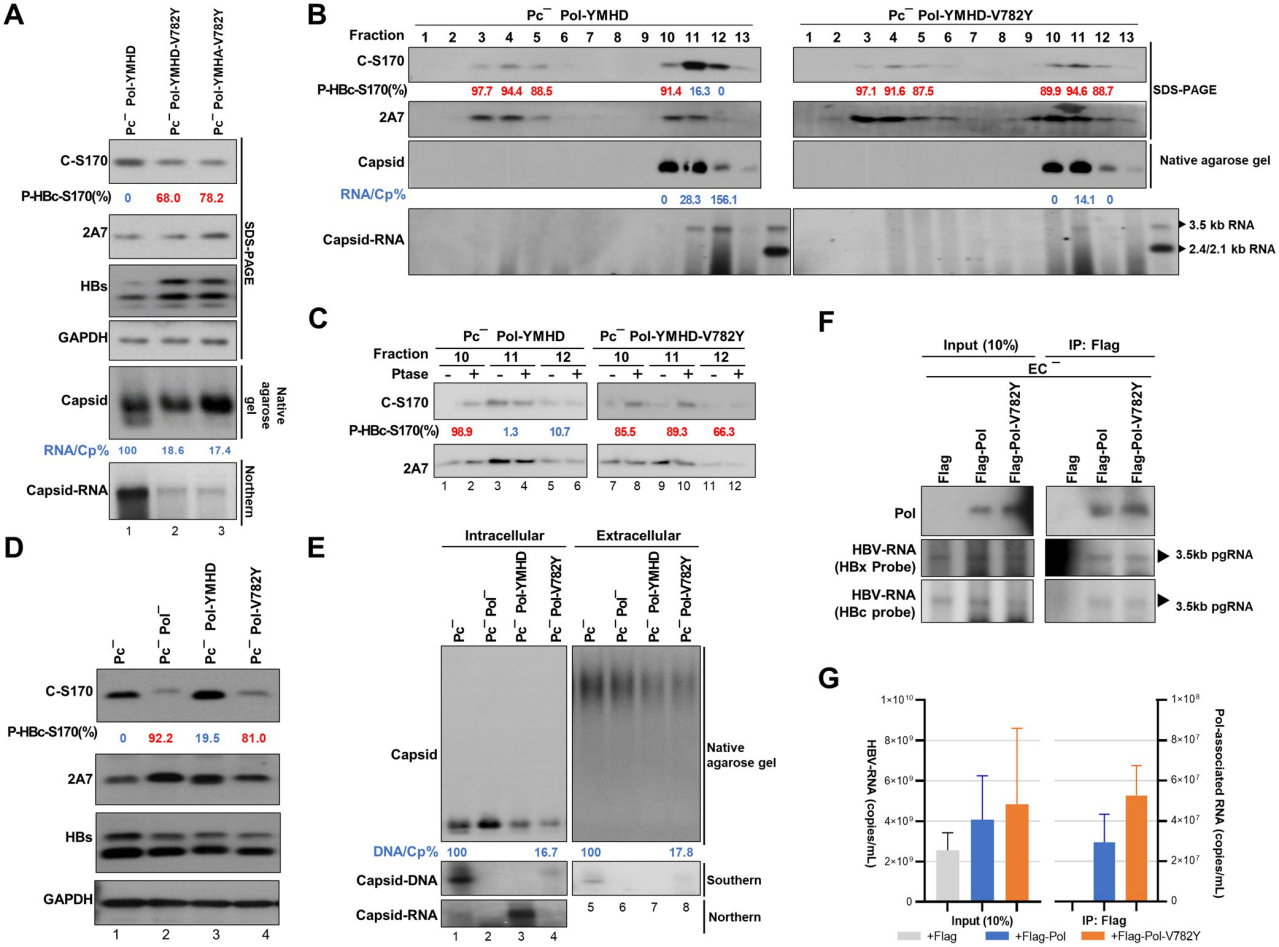
The dephosphorylation of HBc contributes to complete viral RNA encapsidation. **(A)** HBV pgRNA encapsidation of the Pol-V782Y mutant replicons. Huh7 cells were transfected with the indicated HBV replicons for 48 hr, and the phosphorylation patterns were analyzed by immunoblotting to compare the signals of C-S170 Ab with 2A7 Ab. Capsids were analyzed by native agarose gel and capsid RNA was extracted and analyzed by Northern blotting. The P-HBc-S170% of indicated experimental set was determined by $1-\frac{adjusted\;C-S170\;signal\;\left(exp.set\right)}{adjusted\;C-S170\;signal\;\left(Pc^{-}Pol-YMHD\right)}$. **(B)** Sucrose density gradient for HBc dimers/multimers and capsids of the Pol-V782Y mutant replicon. Huh7 cells were transfected with indicated HBV replicons for 48 hr prior to sucrose density gradient analysis (for separation of HBc dimers/multimers and capsids) and analyzed by immunoblotting. Capsid RNA was extracted and analyzed by Northern blotting, with the RNA/Cp% as indicated in fractions 10–12. The phosphorylation patterns were analyzed by immunoblotting. The P-HBc-S170% of indicated fractions was determined by $1-\frac{adjusted\;C-S170\;signal\;\left(indicated\;fraction\right)}{adjusted\;C-S170\;signal\;\left(Pc^{-}Pol-YMHD\;fraction\;12\right)}$. **(C)** The phosphorylation status of the capsid-containing fractions as indicated was validated by the AP assay. The P-HBc-S170% of indicated fractions was determined by $1-\frac{adjusted\;C-S170\;signal\;\left(w/o\;Ptase\;treat\right)}{adjusted\;C-S170\;signal\;\left(w/\;Ptase\;treat\right)}$. **(D)** HBc phosphorylation in Pol-V782Y replicon. Huh7 cells were transfected with indicated HBV replicon constructs for 24 hr. The phosphorylation pattern was analyzed by immunoblotting. The P-HBc-S170% of indicated experimental set was determined by $1-\frac{adjusted\;C-S170\;signal\;\left(exp.set\right)}{adjusted\;C-S170\;signal\;\left(Pc^{-}\right)}$. Hyperphosphorylated HBc-S170 (>50%) is marked in red and hypophosphorylated HBc-S170 (<50%) is marked in blue. **(E)** Capsids and virion DNA content in Pol-V782Y replicon. Huh7 cells were transfected with indicated HBV replicon constructs for 24 hr. Virions were precipitated using sucrose cushion centrifugation. Capsids and virions were resolved by native agarose gel. Capsid DNA extracted from both capsids and virions were analyzed by Southern blotting. Capsid RNA extracted from cell lysates were analyzed by Northern blotting. **(F and G)** RNA-binding ability of Flag-Pol-V782Y. Huh7 cells were transfected with EC^-^ replicon (a precore and core prestop mutant) and Flag-Pol constructs as indicated for 24 hr. Pol-interacting HBV RNA was isolated by immunoprecipitation with Flag-Ab. With equivalent Pol to be immunoprecipitated, the Pol-interacting RNA was extracted and analyzed by Northern blotting (using HBx and HBc probes) and qRT-PCR analysis.

Previous studies of the HBc ARD have suggested that HBc phosphorylation reduces the positive charge of HBc and impairs the encapsidation of pgRNA [18]. To examine the effect of HBc dephosphorylation by PP1 on pgRNA packaging, we introduced the YMHA or YMHD mutation into the Pol-V782Y mutant replicon to arrest HBV replication at the stage when pgRNA-containing capsids accumulate. The results showed that HBc-Ser170 remained highly phosphorylated in both replicons, but the amount of RNA packaged within the capsids was markedly reduced (Fig 6A, lanes 2, 3 vs. lane 1). To further confirm the ability of Pol-V782Y to reduce the formation of genome-containing capsids, we conducted sucrose density gradient for separation of HBc incomplete and complete capsids. The results showed that Pol-V782Y reduced the dephosphorylation of HBc in fractions containing RNA-capsids (Fig 6B and 6C, fractions 11–12) and impeded the enclosing of RNA into capsids (Fig 6B, fractions 11–12). Furthermore, unlike that in the Pc^-^ replicon, HBc-Ser170 in the Pc^-^ Pol-V782Y replicon failed to undergo dephosphorylation (Fig 6D, lane 4), and the amount of encapsidated DNA in both capsids and virions was diminished (>80%) compared to that in the Pc^-^ replicon (Fig 6E, lane 4 vs. 1; lane 8 vs. 5). To exclude the potential impact of Pol-V782Y on pgRNA binding, we isolated viral RNA bound to Flag-tagged Pol-WT or Pol-V782Y through co-IP using Flag-Ab. The results showed no difference in binding to the 3.5 kb pgRNA between WT and mutant Pol for Northern blotting and qRT-PCR (Fig 6F and 6G).

These results provide evidence supporting the crucial role of Pol and a specific residue (Pol-Val782) in regulating HBc dephosphorylation and RNA enclosure into complete capsids during HBV replication via the recruitment of the phosphatase PP1.

## Discussion

In this study, we investigated the ability of the HBc phosphorylation cascade to regulate the HBV life cycle, with a particular focus on the role of HBc hypophosphorylation in RNA encapsidation. Our results revealed dynamic changes in the HBc-Ser170 phosphorylation pattern during the progression of viral replication. The correlation between HBc-Ser170 dephosphorylation and the intricate RNA packaging process suggests a role for Pol in facilitating HBc-Ser170 dephosphorylation during pgRNA encapsidation. In line with this, we demonstrated that Pol recruits the phosphatase PP1 to dephosphorylate HBc-Ser170, which is dependent on pgRNA through the epsilon RNA signal, even in the absence of HBc.

**Fig 7:**
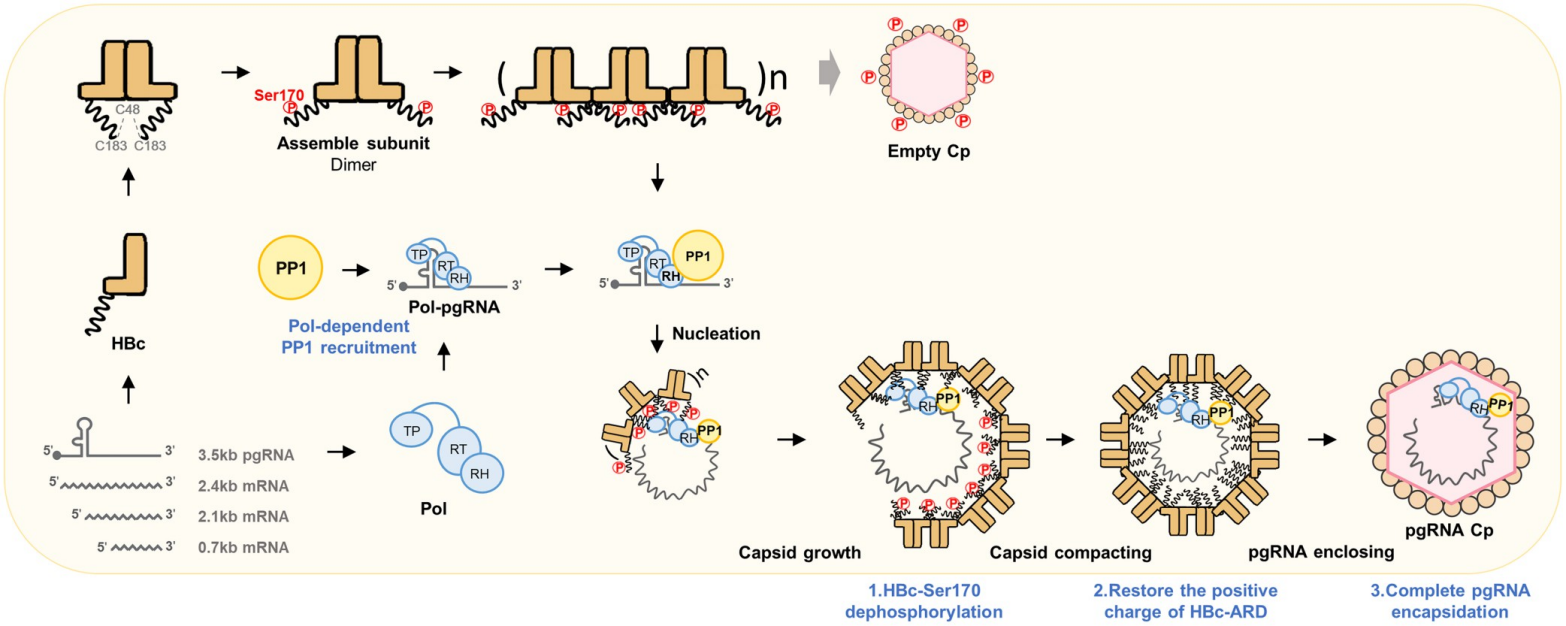
Schematic summary of the coordinated regulation of HBc-Ser170 phosphorylation and dephosphorylation in HBV replication cycle. HBc exists in a hyperphosphorylated state as a dimeric intermediate. The stable phosphorylated HBc intermediate interacts with Pol-pgRNA to initiate nucleation, which directs subsequent capsid growth. During capsid growth, Ser170 residue in HBc is dephosphorylated through the Pol-Val782-recruited phosphatase PP1, dependent on pgRNA via the epsilon RNA signal. This dephosphorylation event restores the positive charge of the HBc ARD, facilitating the compaction and maturation of nucleocapsids.

Thus, our study elegantly elucidates the sequence of events in pgRNA packaging. First, Pol-pgRNA recruits PP1 to form a Pol-pgRNA-PP1 complex. Once this complex binds to the incomplete capsids composed of phosphorylated HBc, PP1 dephosphorylates HBc, which is essential for the subsequent packaging of the Pol-pgRNA-PP1 complex to form compacted capsids to complete the RNA packaging step. The details are summarized in Fig 7. This model aligns with a biphasic model of pgRNA packaging, with an early nuclear phase and a later cytoplasmic phase in the viral life cycle [43], raising the possibility of spatiotemporal regulation of pgRNA encapsidation. The nuclear phase of pgRNA packaging likely corresponds to the early phase of HBV infection. Our findings, demonstrating Pol-mediated PP1 recruitment and HBc dephosphorylation in the cytoplasm, potentially within late endosomes/MVBs, may represent the later cytoplasmic phase of pgRNA packaging and nucleocapsid maturation.

Although our study has clarified the role of Pol-dependent HBc dephosphorylation in the formation of mature HBV nucleocapsids, it is worth noting that previous studies using HBc non-phosphorylatable (Ser to Ala) mutants have demonstrated that phosphorylation of Ser residues at 162 and 170 of HBc is required for the packaging of pgRNA. [20,44], in contrast with our findings. However, this disparity could be reconciled by considering the importance of HBc phosphorylation in the early stage of HBc assembly, with dephosphorylation becoming crucial in the later stages, as discussed in more detail below.

Firstly, it has been proposed that phosphorylated HBc-CTD helps to prevent non-specific RNA packaging because it carries a negative charge [45,46]. At the same time, phosphorylated HBc-Ser170 (and/or HBc-Ser162) also plays a key role in the recruitment of Pol-pgRNA complex, which acts to nucleate capsid assembly and is critical for accumulating the first few subunits of the growing capsid as well as the formation of an incomplete RNA-filled capsids [47,48]. The underlying mechanism may be that phosphorylation of HBc in dimeric and multimeric intermediates helps open the locked CTD of HBc by preventing disulfide bond formation between Cys48 and Cys183 in the dimers [49]. That facilitates the interaction between HBc and the Pol-pgRNA complex and the formation of an incomplete RNA-filled capsid. However, the hyperphosphorylated HBc-CTD carries a negative charge, which may also reduce subsequent pgRNA packaging, leading to incomplete RNA encapsidation [18]. Based on our observations, Pol-pgRNA in incomplete capsids may initiate dephosphorylation of HBc-Ser170 through Val782-mediated PP1 recruitment. This dephosphorylation event restores the positive charge of the HBc ARD and promotes nucleocapsid maturation and compaction (the enclosure step). Thus, our study provides an explanation for the previous observation that the substitution of S to E/D in HBc-Ser170 (especially with the E/D substitution of other Ser residues in CTD) significantly reduces pgRNA packaging [19,20]. Since our C-S170 Ab is specific only for the non-phosphorylated Ser170-containing epitope, further studies may be needed to investigate the effect of Pol-recruited PP1 on other putative phosphorylation sites in HBc CTD.

Therefore, Pol could have dual functions in the RNA encapsidation process: the binding of pgRNA to the HBc dimer/multimer intermediate (nucleation) [30,32,41] and the recruitment of PP1 to dephosphorylate HBc for complete compaction of the nucleocapsid (enclosure). Consistently, recent cryo-EM studies have revealed discernible interior structural differences between the phosphorylated and dephosphorylated forms of the HBc CTD with pgRNA. The interior space adopts a more complex conformation with relatively strong density in phospho-mimic capsids, while it exhibits an icosahedral conformation and weak density, representing native HBV virions, in dephosphorylated capsids [11,50].

In parallel to the SARS-CoV-2 N protein, the phosphorylation changes of HBc might result in the formation of two distinct functional bimolecular condensates. Phosphorylation of the SARS-CoV-2 N protein contributes to the formation of a liquid-like condensate, consisting of HBc dimers or multimers, at the onset of capsid formation. The dephosphorylated N protein undergoes compaction along with viral RNA into gel-like condensates [51,52]. Importantly, our study focused primarily on the later stages of pgRNA encapsidation, and future research should explore the potential specific functions of HBc phosphorylation in the early stages of encapsidation and their relationship with phase separation.

The rationale for designing Pol-V782Y in this study is based on the Pol-V747Y mutation used by Dr. Marion’s team in the DHBV study [33]. Pol-V747 in DHBV is a conserved site in most hepadnaviruses and is equivalent to Pol-V782Y in HBV. In Dr. Marion’s study, this mutation did not affect Pol function to initiate 1st-DNA synthesis. Instead, the Pol-V747Y mutation retains limited RNase H function and caused viral replication to halt at the DNA-RNA hybrid stage. However, our data showed that only a minimal amount of pgRNA is packaged, resulting in capsids containing a very small amount of DNA, which complicates further analysis of DNA synthesis in this context. Whether the small amount of DNA and RNA in the capsids exist as DNA-RNA hybrid form and whether the capsids remain incompletely compacted due to phosphorylation of HBc warrants further investigation. Our study, using Pol-V782Y in HBV, identified for the first time its critical role in recruiting PP1 phosphatase to dephosphorylate HBc-Ser170, which is also worth testing in DHBV or other hepadnaviruses. Additionally, the role of HBc dephosphorylation in viral DNA synthesis is debated. While earlier studies deemed it essential [19,20], recent findings suggest that DNA synthesis can proceed without dephosphorylation [41]. Future studies should compare DNA synthesis between wild-type and dephosphorylation-deficient HBV mutants to clarify the impact of HBc dephosphorylation.

Both PP1 and PP2A play important roles in HBV replication [26,27]. Our results suggest that PP1 is involved in the dephosphorylation of HBc-Ser170 during RNA packaging. The use of the Pol-V782Y mutant (in either the WT or YMHD mutant replicon), which retains two major functions of Pol for encapsidation (formation of Pol-pgRNA complexes and interaction with HBc) but impairs PP1 recruitment, confirmed the crucial role of Pol in mediating the dephosphorylation process to complete RNA encapsidation. The significant decrease in the number of complete virions observed with the Pol-V782Y mutant further emphasizes the importance of HBc-Ser170 dephosphorylation by PP1 in the production of infectious virions. Consistent with this, Prof. Guo’s study also showed that RNA encapsulation in HBV replicon transfected cells was reduced when PP1 activity was inhibited, by using PP1 inhibitors or by knocking down PP1 expression [26]. However, the involvement of other cellular factors affected by PP1 knockdown cannot be excluded.

An alternative hypothesis proposes intrinsic phosphatase activity within the Pol RNase H domain [42]. However, we did not identify any characteristic sequence motifs of serine/threonine-specific phosphatases (PPs) [53] within this region. Instead, our IP-Mass spectrometry and structural analysis identified Pol-Val782 as a critical residue for the Pol-PP1 interaction. This interaction has been independently confirmed in a recently published research, albeit not through the Val686 and Val698 residues [42]. Further investigation into other Pol residues potentially involved in PP1 recruitment, such as Tyr781 and a.a. 814–819, as predicted by AlphaFold (Fig 5C), will be valuable for refining our understanding of this interaction.

In summary, our findings highlight the role of Pol-mediated PP1 recruitment and subsequent HBc-Ser170 dephosphorylation in promoting RNA encapsidation and compaction of the nucleocapsid. Inhibition of host PP1 may hinder viral capsid assembly, and this strategy can be combined with current CpAMs to enhance potency. These insights significantly contribute to understanding the mechanism of HBV replication and may pave the way for the development of novel antiviral strategies targeting this regulatory pathway.

## Materials and methods

### Cell culture

The Huh7 cell line was obtained from the Bioresource Collection and Research Center (BCRC, Taipei, Taiwan) and maintained in DMEM (Gibco, Carlsbad, CA) supplemented with 10% fetal bovine serum (FBS) (Biological Industries, USA) at 37°C in a 5% CO_2_ incubator. For transfection, Huh7 cells were transfected with plasmid DNA using Lipofectamine 2000 (Invitrogen, CA) following the manufacturer’s instructions. The cell lysates were obtained with NET lysis buffer (50 mM Tris, pH 8.0; 1 mM EDTA; 100 mM NaCl; and 0.5% NP40) 24–48 hr after transfection.

### Western blotting

Western blotting was performed as previously described [54]. Briefly, cell lysates were harvested using NET lysis buffer containing 1× proteinase inhibitor (Roche, Basel, Switzerland) and 1× phosphatase inhibitor (Millipore, MA). Protein samples were mixed with 4× SDS loading dye and denatured for 10 minutes at 95°C. The samples were separated on a 12% SDS-PAGE gel and transferred to a polyvinylidene difluoride (PVDF) membrane. The membrane was blocked with 5% milk in 1× TBST before being incubated with primary Abs at 4°C overnight, followed by incubation with secondary Abs. Antigen-Ab complexes were visualized using Plus-ECL reagents (PerkinElmer Japan) or Femto-ECL reagents (GeneTex, Inc., CA).

### Phos-tag SDS-PAGE

Phos-tag SDS-PAGE was performed as previously described [46]. Briefly, cell lysates were harvested using NET lysis buffer containing 1× proteinase inhibitor (Roche, Basel, Switzerland) and 1× phosphatase inhibitor (Millipore, MA). Protein samples were mixed with 4× SDS loading dye and 1 mM MnCl_2_, then denatured for 10 minutes at 95°C. The samples were separated on a 15% Phos-tag SDS-PAGE gel [75 μM Phos-tag acrylamide AAL-107 (Wako Pure Chemical Corporation, Japan), 150 μM MnCl_2_]. Before transfer to PVDF membrane, the gel was soaked in the transfer buffer containing 1 mM EDTA for 15 minutes with gentle agitation.

### Antibodies

The C-S170 Ab was generated by immunizing rabbits with synthetic peptide (PRRRRSQ-S-PRRRR) containing the Ser170 obtained from LTK bio Laboratories Inc. (Taiwan). The 2A7 (anti-HBcAg) Ab was generously provided by Professor Quan Yuan from Xiamen University (Fujian, China). Abs against PP1, PP1Cβ, PP2ACα, PP2ACβ, GRP78, TGN46, CHMP2A, CD63 and GAPDH were purchased from Genetex (GeneTex, Inc., CA). Abs against PP1Cα, p-GSK3β, GSK3β, p-CREB, and CREB were purchased from Cell Signaling Technology (CST, MA). Abs against Pol (2C8) and Rab7 were obtained from Santa Cruz Biotechnology Inc. (Santa Cruz, CA).

### Plasmids

The pAAV/HBV1.2 HBV replicon plasmid contains a 1.2-fold over-length HBV genotype A genome (nt 1400-3182/1-1987) [55] and was used as WT replicon plasmid for the construction of different mutant replicon constructs that stop at different replication stages. To generate the HBc/precore-null (EC^-^) replicon, a prestop codon was introduced into the HBc/precore open reading frame. The ε-B1G mutant replicon, designed to abolish Pol-pgRNA interaction, was created by introducing a C-to-G mutation at nucleotide 1860 (corresponding to the first nucleotide of the ε bulge) [41]. All specific genetic mutations used in this study (listed in S1 Fig) were introduced into each mutant replicon constructs, in specific domains of individual viral proteins, by site directed mutagenesis according to the manufacturer’s instructions (QuikChange Lightning Site-Directed Mutagenesis Kit, Agilent, CA).

The CMV-HBc expression plasmid was constructed by amplifying a PCR fragment containing the HBc coding region using pAAV/HBV1.2 as the template. The PCR product was digested with BamHI and NotI, cloned and inserted into the corresponding sites of pcDNA3.1 (+). The pCMV-Pol and pCMV-Pol-V782Y plasmids were generated from a wild-type polymerase expression vector, pMT-Pol [56]. A PCR fragment containing the coding region of the polymerase was amplified, digested with NheI and NotI, cloned and inserted into the respective sites of pcDNA3.1 (+). To construct pCMV-Flag-Pol and pCMV-Flag-Pol-V782Y, a PCR fragment containing the Pol coding region was amplified from pMT-Pol, digested with HindIII and KpnI, cloned and inserted into the corresponding sites of pFLAG-CMV-2. All constructs were validated by sequencing the entire inserts to confirm their authenticity and rule out any second site mutations. The sequences of all primer sets and restriction enzymes used in this study are available upon request.

### ELISA

To quantify the binding specificity of C-S170 Ab with non-phosphorylated HBc-Ser170, a flat-bottomed 96-well plate was coated with 100 ng of various peptides, including the non-phosphorylated and phosphorylated peptides covering Ser150, Ser162 and Ser170, and then the C-S170 Ab was added to the wells. For the competition of various peptides to C-S170 Ab, the 96-well plate was coated with 100 ng of non-phosphorylated HBc-Ser170 peptide and then a preincubated mixture containing 100 ng of peptide and the C-S170 Ab was added to the wells. After incubation with rabbit IgG conjugated with HRP, TMB substrate and a stop solution were added. The absorbance at a wavelength of 450 nm (A450) with a reference wavelength of 650 nm was measured using a microplate reader [57]. GraphPad Prism 5 software was used to generate bar graphs and calculate *Kd* values, binding percentages, and inhibition percentages. The sequences of all peptides used in this study were listed in Fig 1A.

### *In vitro* dephosphorylation analysis (alkaline phosphatase assay)

Cell lysates derived from NET lysis buffer were mixed with a mixture of Alkaline phosphatase (FastAP) (Thermo Scientific, CA) and the manufacturer’s buffers, and the mixture was incubated at 37°C for 2 hr. Phosphatase-treated cell lysates were analyzed using Western blotting.

### Determination of the phosphorylation status of HBc Protein

The percentage of HBc Ser170 phosphorylation (P-HBc-S170%) expressed by indicated replicon constructs (experimental sets) was determined by comparing the adjusted C-S170 Ab signal (adjusted with 2A7 Ab signal, the total amount of HBc protein) with the adjusted signal of the control reHBc or HBc expressed by indicated replicon constructs (as dephosphorylated HBc control in each set of experiments), calculated as *$1-\frac{adjusted\;C-S170\;signal\;\left(exp.set\right)}{adjusted\;C-S170\;signal\;\left(deP\;control\right)}$*.

Moreover, the P-HBc-S170% of selected experimental replicon constructs was further validated by AP analysis as mentioned above, which was determined by comparing the C-S170 Ab signal (adjusted with 2A7 Ab signal, the total amount of HBc protein) with and without AP treatment. P-HBc-S170% was then calculated as $1-\frac{C-S170\;signal\;\left(w/o\;Ptase\;treat\right)\times 2A7\;ratio\;\left(\frac{w/\;Ptase\;treat}{w/o\;Ptase\;treat}\right)}{C-S170\;signal\;\left(w/\;Ptase\;treat\right)}$, and expressed as $1-\frac{adjusted\;C-S170\;signal\;\left(w/o\;Ptase\;treat\right)}{adjusted\;C-S170\;signal\;\left(w/\;Ptase\;treat\right)}$ in text or figure legends.

### Detection of the HBV capsid and viral DNA in the capsids (Southern blotting)

The capsid DNA was detected by two methods. For Southern blotting assay, cell lysates derived from NET lysis buffer were treated with micrococcal nuclease (New England BioLabs, MA) to digest cellular nucleic acids. HBV nucleocapsid-associated DNA was extracted using the phenol-chloroform extraction method. Southern blotting analysis was performed to detect HBV DNA using a 1.5% agarose gel, and the DNA was transferred to a nylon membrane (Hybond-N, GE Healthcare, IL).

For native agarose gel assay, cell lysates derived from NET lysis buffer were separated by 1.5% native agarose gel electrophoresis in 1× TBE buffer and subsequently transferred to PVDF membranes by capillary transfer. The membranes were blocked and incubated with either a rabbit polyclonal anti-HBc Ag Ab from Dako (Dako Cytomation Co., CA) or 2A7 (anti-HBcAg) primary Ab. After incubation with the appropriate secondary Ab, the signals were detected using Plus-ECL reagents (PerkinElmer, Japan). To detect viral DNA in the capsids, the membranes were incubated with denaturing buffer (0.2 M NaOH, 1.5 M NaCl) followed by neutralizing buffer (0.2 M Tris-HCl, 1.5 M NaCl, pH 7.5). Southern blotting analysis was performed using digoxigenin (DIG)-labeled probes as previously described [58]. The DIG-HBx probe was used to detect HBV DNA.

### Detection of HBV RNA in capsids (Northern blotting)

Cell lysates derived from NET lysis buffer were treated with micrococcal nuclease (New England BioLabs, MA) to digest cellular nucleic acids. HBV nucleocapsid-associated RNA was extracted using the phenol-chloroform extraction method. Northern blotting analysis was performed to detect purified RNA using a 1.5% agarose gel containing formaldehyde in MOPS buffer, and the RNA was transferred to a nylon membrane (Hybond-N, GE Healthcare, IL). The DIG-HBx probe and DIG-HBc probe were used to detect HBV RNA.

### Precipitation of HBV viral particles

Supernatant from HBV replicon transfected Huh7 cells was used to precipitate HBV viral particles. Sucrose density gradients were prepared in 1× phosphate buffered saline (PBS). The 9 mL supernatant was layered on 1 mL of 20% sucrose for sucrose cushion centrifugation and then subjected to ultracentrifugation at 25,000 rpm for 16 hr at 4°C. The viral particles were resuspended in 1×PBS.

### Sucrose density gradient for separation of different HBc conformations

A sucrose density gradient was used to separate different conformations of HBc. Sucrose density gradients were prepared in gradient buffer (50 mM Tris, pH 7.5; 50 mM NaCl; and 1 mM EDTA, pH 8.0). The cell lysates derived from NET lysis buffer were layered sequentially from 60% to 15% sucrose for sucrose density gradient centrifugation and then subjected to ultracentrifugation at 40,000 rpm for 6 hr at 4°C.

### Sucrose density centrifugation for separation of intracellular organelles

A sucrose density gradient was used to separate intracellular organelles [39]. Huh7 cells were lysed with IB buffer (0.25 M sucrose, 10 mM HEPES, and 10 mM EDTA). Cytoplasmic proteins and membrane organelles were obtained by centrifuging at 1,000 × g at 4°C for 10 minutes, and the supernatant was collected. The cell lysates derived from IB buffer were layered sequentially from 1.5 M to 1 M sucrose for sucrose density gradient centrifugation. The sucrose density gradient was subjected to ultracentrifugation at 28,000 rpm for 4 hr at 4°C. Organelles in each fraction were verified with Ab specific for each organelle indicator protein.

### Phosphatase gene knockdown

Recombinant shRNA-expressing lentiviruses were generated by co-transfecting 293T cells with the lentiviral vector pLKO.1 carrying shRNA, pCMV-dR8.91 (a packaging vector), and pMD.G (a VSV-G expression vector) using TransIT-LT1 (Mirus Bio., WI). The lentiviruses were harvested 48 and 72 hr after transfection. The TRC shRNA stocks used in the study were provided by the National RNAi Core Facility (Academia Sinica, Taipei, Taiwan). For the knockdown of PP1Cα, shRNA clones TRCN0000002455 and TRCN0000318526 were used. For the knockdown of PP1Cβ, shRNA clones TRCN0000338328 and TRCN0000338386 were used. For the knockdown of PP2ACα, shRNA clones TRCN0000002483 and TRCN0000002486 were used. For the knockdown of PP2ACβ, shRNA clones TRCN0000002505 and TRCN0000002506 were used. Luciferase and LacZ shRNA were used as the negative control. Huh7 cells were infected with the lentivirus by addition to the medium for 16 hr, after which the cells were transfected with the Pc^-^ replicon for 24 hr.

### Coimmunoprecipitation (Co-IP)

For Flag-Pol co-IP, cell lysates obtained from NET lysis buffer were incubated with Anti-FLAG M2 Affinity Gel (Sigma–Aldrich, MA) in IP buffer (20 mM Tris pH 7.4, 50 mM NaCl, 1 mM EDTA pH 8.0, 1 mM EGTA pH 8.0, 0.1% NP40, 1 mg/mL BSA, 10% glycerol) containing 1× protease inhibitor and 1× phosphatase inhibitor for 16 hr. For other protein co-IP, cell lysates obtained using NET lysis buffer were incubated with the indicated Ab in IP buffer containing 1× protease inhibitor and 1× phosphatase inhibitor for 16hr. Subsequently, the samples were incubated with Protein G (Sigma-Aldrich, MA) for 4 hr. The immunoprecipitated samples were then washed with IP buffer and eluted in 1× SDS-PAGE sample buffer (50 mM Tris, pH 6.8, 2% SDS, 3.75% β-mercaptoethanol, 10% glycerol, and 0.01% bromophenol blue) for Western blotting analysis. For RNA co-IP, cells were lysed with RNA-IP buffer (50 mM Tris pH 8.0, 1 mM EDTA, 100 mM NaCl, 0.5% NP40). The cell lysates were then incubated with Anti-FLAG M2 Affinity Gel in RNA-IP buffer containing 10 mM β-mercaptoethanol, 2 mM DTT, 250U/mL RNase Out (Thermo Fisher Scientific, CA), 1× protease inhibitor and 1× phosphatase inhibitor. The immunoprecipitated samples were washed with RNA-IP buffer and then divided into 2 parts. One-tenth of each immunoprecipitated sample was eluted in sample buffer for Western blotting analysis, while the remaining nine-tenths were eluted in REzol (Invitrogen, CA) to extract RNA for Northern blotting and qRT-PCR analysis.

### Quantitative PCR (qPCR) and quantitative reverse transcription PCR (qRT-PCR)

The HBV capsids was treated with micrococcal nuclease (New England BioLabs, MA), and then HBV DNA was extracted by the phenol-chloroform extraction method. Pol-associated RNA, isolated by co-IP with Pol, was reverse transcribed using SuperScript III Reverse Transcriptase System (Thermo Fisher Scientific, CA). Quantification of HBV DNA and reverse transcription products was performed using FastStart DNA SYBR Green on a LightCycler 1.5 (Roche Diagnostics, Basel, Switzerland), following the manufacturer’s instructions. The primers for the HBV genome and the reverse transcribed products were HBx-F: 5’- CCGATCCATACTGCGGAAC-3’ and HBx-R: 5’- GCAGAGGTGAAGCGAAGTGCA-3’, respectively, and were quantified with the protocol as described previously [59].

### Prediction of protein-protein interactions with AlphaFold2

Sequences were predicted using AlphaFold Colab (https://colab.research.google.com/github/deepmind/alphafold/blob/main/notebooks/AlphaFold.ipynb) with the default parameters. A multimer model was generated for the prediction of protein-protein interactions [60]. Prediction graphics and analyses were performed with UCSF ChimeraX [61].

## Supporting information

S1 Fig Schematic representation of different HBV replicon constructs, introducing specific mutations in different structural domains of individual viral proteins. (Upper panel) The four overlapping open reading frames (ORFs) encoded by the HBV genome (genotype A) are highlighted in different colors (orange: precore/HBc; blue: Pol; green: HBs; gray: HBx); the specific structural domains in each ORF are also highlighted accordingly. (Lower panel) Illustration of genetic mutations introduced in each mutant replicon constructs, in specific domain of individual viral protein. The position of each mutation relative to the wild-type HBV genomic sequence and a.a. codon is highlighted, and the nucleotide and a.a. changes are marked with different colors according to the associated viral protein. #, The Pol-Y65D mutation in genotype A HBV as shown in the figure corresponds to Pol-Y63D in other genotypes of HBV viruses.
(TIF)

S2 Fig Validation of the enrichment of specific replication intermediates by different HBV replicon constructs. **(A)** Schematic representation of distinct replication intermediates in HBV replication cycle. **(B)** Huh7 cells were transfected with indicated HBV replicon constructs for 24 hr. The capsids and capsid DNA were analyzed by native agarose gel electrophoresis and Southern blotting, and the capsid RNA was analyzed by Northern blotting. **(C)** Huh7 cells were transfected with the PC^-^ replicon construct for 120 hr. Virions in the supernatant were precipitated by cushion for subsequent sucrose density gradient to separate the empty and complete virions. The virions and naked capsid were analyzed by SDS-PAGE and native agarose gel (upper panel), and capsid DNA in each fraction was quantified by qPCR analysis (lower panel). Fraction 5 (empty virions) and fractions 6–7 (genome-containing virions) were processed to analyze the phosphorylation status of HBc-Ser170 by AP assay (data in Fig 2C).
(TIF)

S3 Fig PP1 is encapsidated into the genome-containing nucleocapsids. **(A)** Huh7 cells were transfected with indicated HBV replicon constructs for 48 hr, followed by sucrose density gradient analysis to separate HBc dimers/multimers and capsids (as indicated in Fig 3C). The fractions containing empty capsids and genome-containing capsids, fractions 8–10 as indicated, were concentrated using 20% sucrose cushion and processed for immunoblotting analysis. **(B)** Huh7 cells were transfected with indicated HBV replicon constructs for 48 hr. HBc-interacting PP1 and PP1-interacting HBc were immunoprecipitated with Protein G beads and the indicated antibodies. The co-IP samples were processed for immunoblotting analysis.
(TIF)

S4 Fig Predicted model of the Pol-RNase H domain and PP2ACα interaction. Cartoon representation of the structures of Pol-RNase H (blue) and PP2ACα (light yellow). Pol-Val782 is marked in dark blue.
(TIF)

S5 Fig Distribution of cellular compartments in a sucrose density gradient for cell fractionation. Distribution of the indicators for different cellular compartments, as a control for the results shown in Fig 5E. Huh7 cells were cotransfected with HBV replicon and Pol constructs as indicated for 48 hr. Cellular organelles were separated using a sucrose density gradient for cell fractionation and analyzed by immunoblotting, probing with Abs for indicators of different cellular compartments as indicated. Calibrator: an aliquot of lysate from Pc^-^ Pol^-^ LMS^-^ + Pol-V782Y transfected cells.
(TIF)

S6 Fig The pgRNA is essential for Pol-dependent PP1 recruitment and dephosphorylation of HBc-S170. **(A)** Distribution of PP1 in cells expressing the Pol constructs as indicated. Huh7 cells were transfected with Flag-Pol and Flag-Pol-V782Y constructs for 48 hr. Cell compartments were separated using a sucrose density gradient for cell fractionation and resolved by SDS-PAGE. Calibrator: an aliquot of lysate from Flag-Pol transfected cells. **(B)** Distribution of PP1 in cells expressing the HBc-lacking replicons. Huh7 cells were cotransfected with Ec^-^ Pol^-^ replicon and Pol construct for 48 hr. Cell compartments were separated using a sucrose density gradient for cell fractionation and resolved by SDS-PAGE. Calibrator: an aliquot of lysate from Ec^-^ Pol^-^ + Pol transfected cells. **(C)** Validation of the ε-B1G mutant on pgRNA encapsidation. (left panel) Schematic illustration of the epsilon signal of pgRNA. The ε-B1G mutant on the first nucleotide in bulge. (Right panel) Huh7 cells were transfected with indicated HBV replicon constructs for 24 hr. The protein lysate was analyzed by SDS-PAGE, the capsids and capsid DNA were analyzed by native agarose gel electrophoresis, and the capsid RNA was analyzed by Northern blotting. (D) Distribution of PP1 in cells expressing the HBV replicon without interactions between Pol and pgRNA. Huh7 cells were cotransfected with Pc^-^ ε-B1G Pol^-^ replicon and Pol constructs for 48 hr. Cell compartments were separated using a sucrose density gradient for cell fractionation and resolved by SDS-PAGE. Calibrator: an aliquot of lysate from Pc^-^ ε-B1G Pol^-^ + Pol transfected cells.
(TIF)

S1 Table Pol-interacting phosphatases identified by IP/mass spectrometry analysis. Pol-interacting phosphatases identified via IP/mass spectrometry. Huh7 cells were cotransfected with the HBV replicon and Flag-Pol constructs as indicated for 24 hr. Pol-interacting proteins were coimmunoprecipitated and eluted via SDS-PAGE for LC-MS analysis. MS data were filtered to identify Pol-interacting phosphatases. # PSMs: Peptide spectrum matches.
(PDF)

S2 Table The numerical data used in all figures. (XLSX)

S1 File Predicted model of the Pol-RNase H domain and PP1Cα interaction. The predicted interaction model of Pol-RNase H domain and PP1Cα using AlphaFold.
(PDB)

S2 File Predicted model of the Pol-V782Y RNase H domain and PP1Cα interaction. The predicted interaction model of Pol-V782Y RNase H domain and PP1Cα using AlphaFold.
(PDB)

S3 File Predicted model of the Pol-RNase H domain and PP2ACα interaction. The predicted interaction model of Pol-RNase H domain and PP2ACα using AlphaFold.
(PDB)
