# Supplementary figures and images for: HBV polymerase recruits the phosphatase PP1 to dephosphorylate HBc-Ser170 to complete encapsidation
Source: PLoS Pathog. 2025 Feb 11;21(2):e1012905. doi: 10.1371/journal.ppat.1012905 (PMC11813143; doi:10.1371/journal.ppat.1012905)

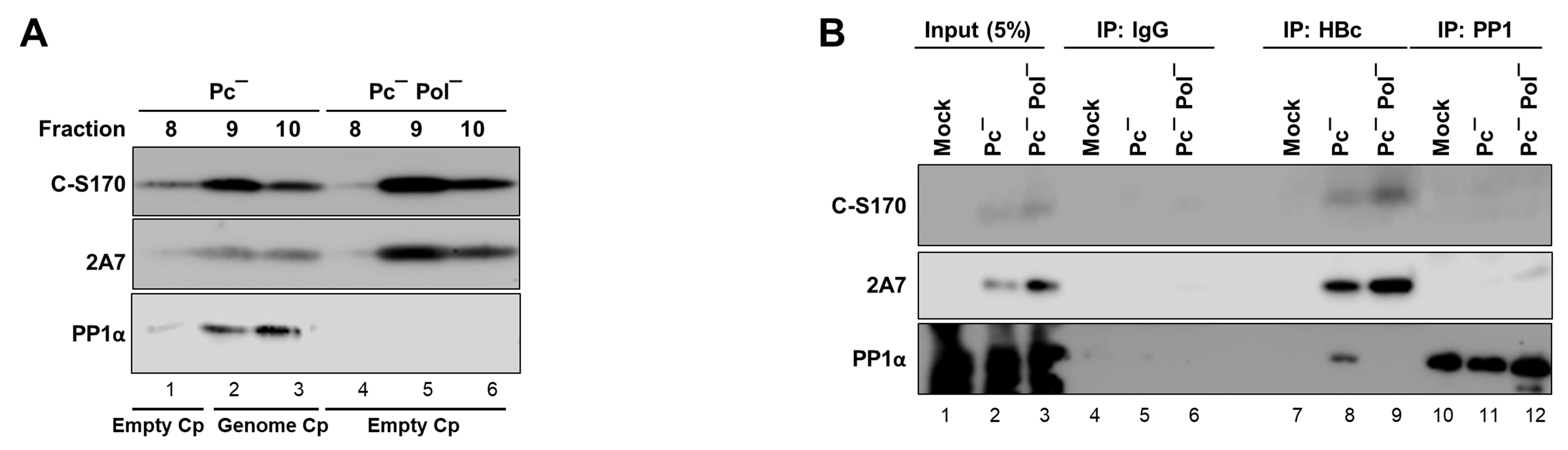

Supplement: S3 Fig — (A) Huh7 cells were transfected with indicated HBV replicon constructs for 48 hr, followed by sucrose density gradient analysis to separate HBc dimers/multimers and capsids (as indicated in Fig 3C). The fractions containing empty capsids and genome-containing capsids, fractions 8–10 as indicated, were concentrated using 20% sucrose cushion and processed for immunoblotting analysis. (B) Huh7 cells were transfected with indicated HBV replicon constructs for 48 hr. HBc-interacting PP1 and PP1-interacting HBc were immunoprecipitated with Protein G beads and the indicated antibodies. The co-IP samples were processed for immunoblotting analysis. (TIF) [file ppat.1012905.s003.tif]

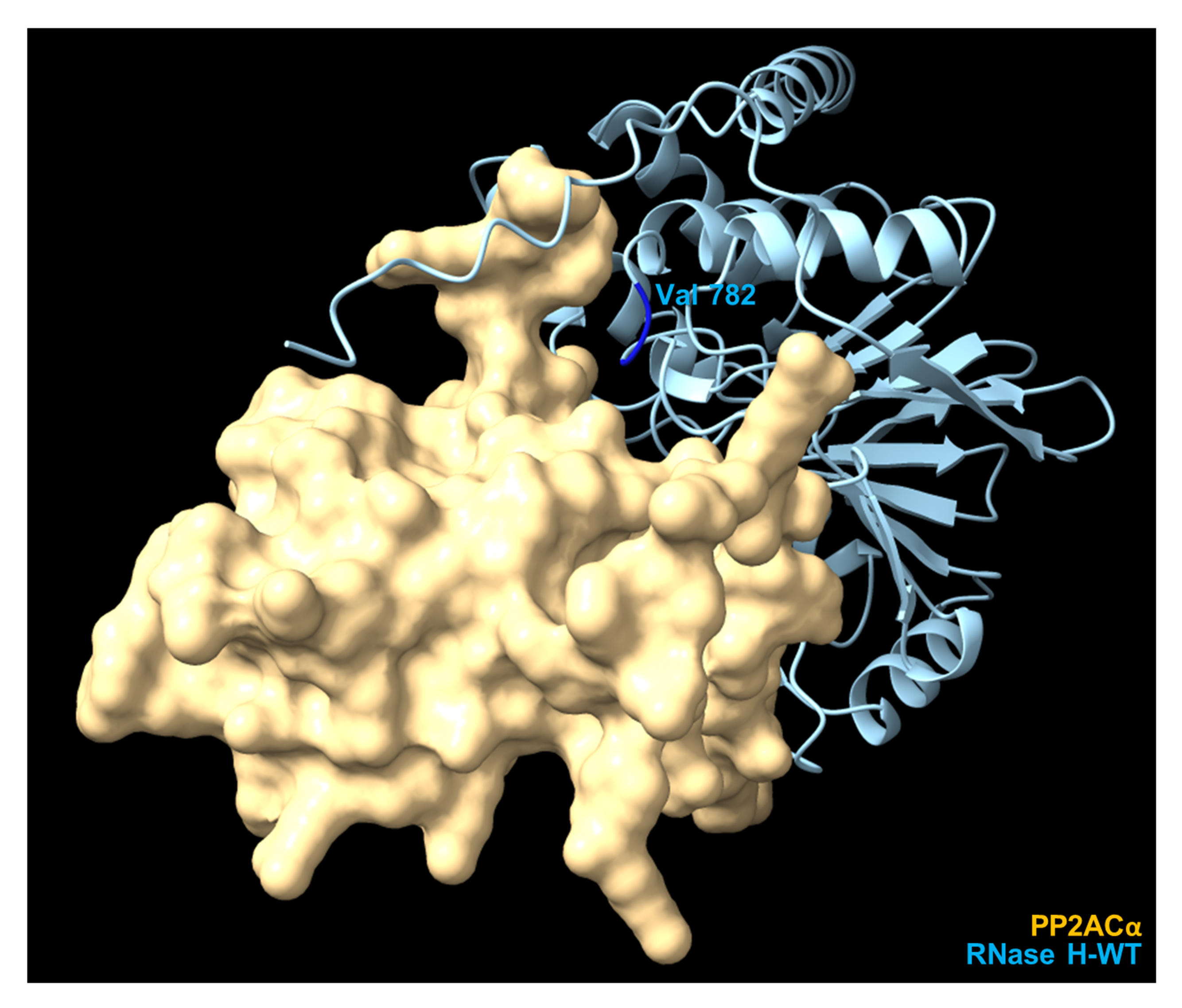

Supplement: S4 Fig — Cartoon representation of the structures of Pol-RNase H (blue) and PP2ACα (light yellow). Pol-Val782 is marked in dark blue. (TIF) [file ppat.1012905.s004.tif]

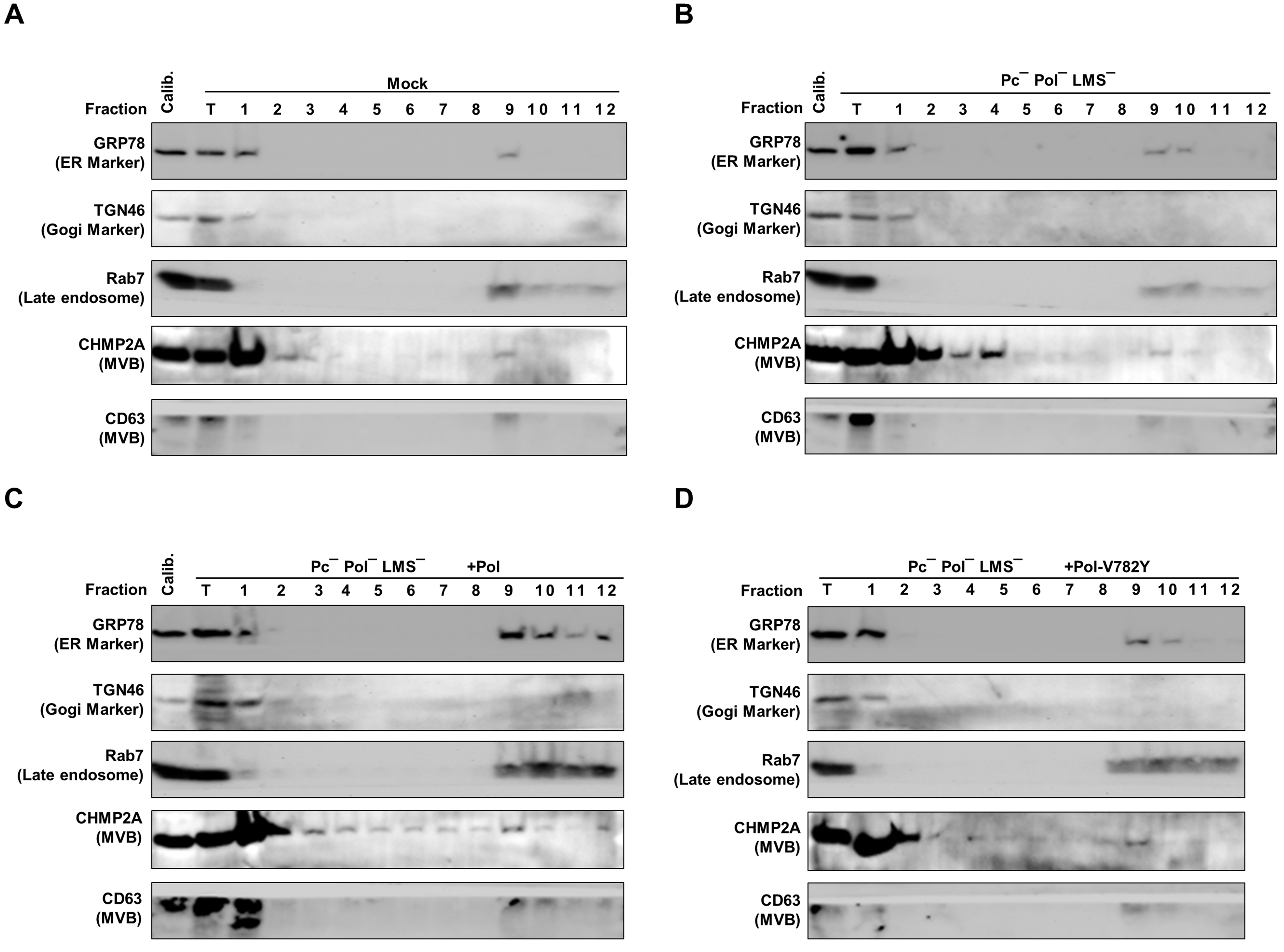

Supplement: S5 Fig — Distribution of the indicators for different cellular compartments, as a control for the results shown in Fig 5E. Huh7 cells were cotransfected with HBV replicon and Pol constructs as indicated for 48 hr. Cellular organelles were separated using a sucrose density gradient for cell fractionation and analyzed by immunoblotting, probing with Abs for indicators of different cellular compartments as indicated. Calibrator: an aliquot of lysate from Pc- Pol- LMS- + Pol-V782Y transfected cells. (TIF) [file ppat.1012905.s005.tif]
