# Supplementary material for: HBV polymerase recruits the phosphatase PP1 to dephosphorylate HBc-Ser170 to complete encapsidation
Source: PLoS Pathog. 2025 Feb 11;21(2):e1012905. doi: 10.1371/journal.ppat.1012905 (PMC11813143; doi:10.1371/journal.ppat.1012905)
Supplement: S1 Table — Pol-interacting phosphatases identified via IP/mass spectrometry. Huh7 cells were cotransfected with the HBV replicon and Flag-Pol constructs as indicated for 24 hr. Pol-interacting proteins were coimmunoprecipitated and eluted via SDS-PAGE for LC-MS analysis. MS data were filtered to identify Pol-interacting phosphatases. # PSMs: Peptide spectrum matches. (PDF) [file ppat.1012905.s007.pdf]

Table S1. Pol-interacting phosphatases identified by IP/mass spectrometry analysis.

Description	Found in Pol			Found in Pol-V782Y			Pol/Pol-V782Y
	Abundances	Coverage[%]	# PSMs	Abundances	Coverage[%]	# PSMs	(Abundance)
**Pol**	67429377	4.89	10	54403378	4.73	10	1.24
**HBc**	2878398	6.01	1	2623369	6.01	1	1.1
**PP1Cα/β**	2388880	3.36	1	0	0	0	-
**PP1Cγ**	2388880	3.1	1	0	0	0	-
**PPM1A**	9697469	5.24	3	4311901	5.24	3	2.25
**PPM1B**	15338095	18.37	6	8719157	5.22	2	1.76
**PP2A-R1A**	3494543	8.66	3	4520082	4.58	2	0.77
**PP2A-R1B**	2219860	4.7	2	3343304	4.7	2	0.66
**PP2A-R2A**	0	0	0	1278921	3.8	1	0
**PPP6-R3**	1779114	0.8	1	0	0	0	-

PSMs: Peptide spectrum matches.
